# Quantitative Profiling of the Human Substantia Nigra Proteome from Laser-capture Microdissected FFPE Tissue*[Fn FN2]

**DOI:** 10.1074/mcp.RA119.001889

**Published:** 2020-03-04

**Authors:** Eva Griesser, Hannah Wyatt, Sara Ten Have, Birgit Stierstorfer, Martin Lenter, Angus I. Lamond

**Affiliations:** ‡ Centre for Gene Regulation and Expression, School of Life Sciences, University of Dundee, Dundee, DD1 5EH, United Kingdom; § Drug Discovery Sciences, Boehringer Ingelheim Pharma GmbH & Co. KG, Biberach an der Riss, Germany

**Keywords:** Tandem mass spectrometry, tissues, quantification, Parkinson's disease, HPLC, FFPE, laser-capture microdissection, substantia nigra, TMT

## Abstract

Laser-capture microdissection (LCM) allows the visualization and isolation of morphologically distinct subpopulations of cells from heterogeneous tissue specimens. In combination with formalin-fixed and paraffin-embedded (FFPE) tissue it provides a powerful tool for retrospective and clinically relevant studies of tissue proteins in a healthy and diseased context. We first optimized the protocol for efficient LCM analysis of FFPE tissue specimens. The use of SDS containing extraction buffer in combination with the single-pot solid-phase-enhanced sample preparation (SP3) digest method gave the best results regarding protein yield and protein/peptide identifications. Microdissected FFPE human substantia nigra tissue samples (∼3,000 cells) were then analyzed, using tandem mass tag (TMT) labeling and LC-MS/MS, resulting in the quantification of >5,600 protein groups. Nigral proteins were classified and analyzed by abundance, showing an enrichment of extracellular exosome and neuron-specific gene ontology (GO) terms among the higher abundance proteins. Comparison of microdissected samples with intact tissue sections, using a label-free shotgun approach, revealed an enrichment of neuronal cell type markers, such as tyrosine hydroxylase and alpha-synuclein, as well as proteins annotated with neuron-specific GO terms. Overall, this study provides a detailed protocol for laser-capture proteomics using FFPE tissue and demonstrates the efficiency of LCM analysis of distinct cell subpopulations for proteomic analysis using low sample amounts.

## Abstract (precis)

Laser-capture microdissection of formalin-fixed and paraffin-embedded (FFPE) tissue is a powerful tool for the analysis of cells present in small cell numbers. It allows retrospective studies of healthy and diseased tissue specimens considering the huge repositories for FFPE tissue. A protocol is described with detailed evaluation of each sample preparation step including protein extraction from unstained and immunohistochemical stained tissue, SP3 digestion and TMT labeling. Finally, the established method was applied to limited sample amounts of human substantia nigra.

## Graphical Abstract

**Figure:**
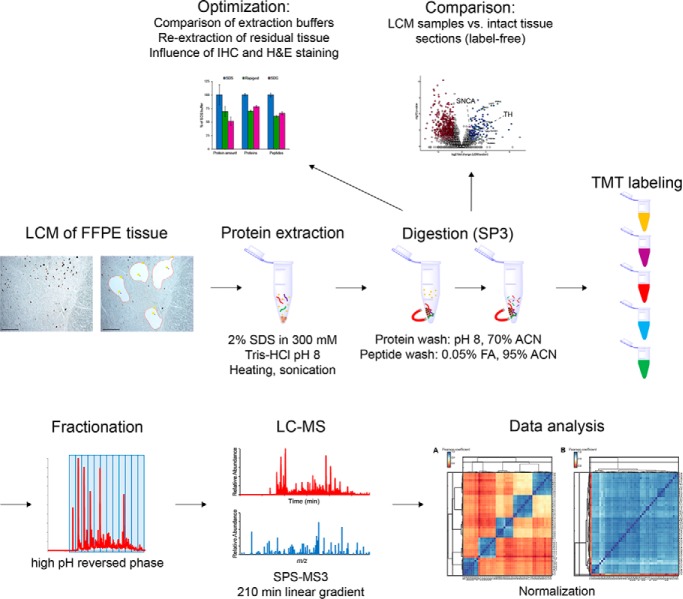

**Highlights**

- A detailed investigation of the protein extraction step from FFPE tissue is shown.
- Acidification during peptide wash increased peptide recovery of the SP3 method.
- LCM of substantia nigra enriched neuron-specific proteins including TH.
- >5,600 proteins were quantified using 3000 cells per sample from substantia nigra.

Formalin-fixed and paraffin-embedded (FFPE)^1^ tissue is a gold mine for retrospective clinical studies considering the huge tissue repositories available worldwide. The samples are not contagious, can be stored for years at room temperature and preserve the structure and morphology of the tissue (1). FFPE is routinely used in pathology departments worldwide to archive tissue specimens. The fixation with formalin preserves the tissue via cross-linking of formaldehyde with nucleic acids, polysaccharides and protein side chains (2). However, these cross-links and modifications with formaldehyde result in poor extraction of proteins and RNA/DNA. Therefore, for subsequent biochemical analysis, the cross-links need to be reversed, which can be achieved via heating (“antigen retrieval”) (2). Variation in the protocols used by different laboratories, regarding fixation times, formalin concentrations, temperature and pH, influence the quality of tissue specimens and influence the efficiency of protein extraction from FFPE tissue (3).

Besides heating of samples, different extraction buffers have been used to achieve efficient protein extraction. These buffers commonly contain Tris and SDS, but also other detergents, such as Rapigest, chaotropes (*e.g.* guanidine-HCl) and organic solvents (*e.g.* trifluoroethanol, acetonitrile) have been used to solubilize proteins from FFPE tissue (1, 2, 4).

Tissue sections are a heterogeneous mix of diverse cell populations present in different cell numbers. Laser-capture microdissection (LCM) is a powerful technique for visualizing and isolating distinct cell subpopulations from heterogeneous tissue in its morphological context. LCM enables enrichment of cells and analysis of protein expression in specific cell subpopulations. For example, using LCM, neuromelanin granules were recently isolated from substantia nigra tissue (5), whereas Drummond *et al.* purified neurons and amyloid plaques from Alzheimer's disease brain tissue (6, 7). However, a challenge for combining LCM with proteomics is the small quantity of isolated cells and correspondingly low levels of proteins, which usually prohibits fractionation of samples and their in-depth analysis. However, multiplexing of samples, via the use of isobaric TMT, can increase total peptide levels and facilitate fractionation (8, 9).

The substantia nigra is an area in the midbrain containing brown-pigmented granules (“neuromelanin”), which are part of dopaminergic neurons. The loss of these neurons in substantia nigra pars compacta is a hallmark of Parkinson's disease (10). Therefore, several studies have already analyzed the nigral proteome and compared samples from healthy donors with diseased ones (11–13). Additionally, studies isolating neuromelanin from substantia nigra tissue have been performed recently (5, 14). Overall, up to 1795 proteins per study were identified. However, these previous studies did not involve sample fractionation to improve the depth of analysis. Additionally, they were performed using frozen tissues, whereas FFPE tissue material offers better tissue morphology allowing a more precise identification and finally isolation of target cells.

In this study, we have developed an optimized protocol to facilitate efficient LCM analysis of FFPE tissue specimens. A thorough investigation of each sample preparation step is provided including the optimization of protein extraction from FFPE tissue by testing different extraction buffers and investigating the influence of immunohistochemical (IHC) and hematoxylin & eosin (H&E) staining on proteins. SDS present in the protein extracts was removed with the SP3 digest method, which was modified to improve protein and peptide recoveries. Thus, acidification of the peptide wash solution could significantly increase the peptide recovery. Protein expression of microdissected samples was compared with intact tissue sections from substantia nigra to evaluate the efficiency of LCM for the purification of small cell populations, which resulted in the enrichment of neuron-specific proteins including tyrosine hydroxylase and alpha-synuclein. The optimized protocol was used to analyze samples containing as few as ∼3000 cells isolated from the substantia nigra, using FFPE tissue. Replicate samples of 15 healthy donors were analyzed in five separate TMT10plex batches giving an overview of the abundance and annotated GO terms of nigral proteins. This study provides a detailed workflow for laser-capture proteomics of samples from FFPE tissue using limited sample amounts and represents the most in-depth proteomic data set for human substantia nigra samples reported to date.

## EXPERIMENTAL PROCEDURES

### 

#### 

##### Tissue Samples and Laser-capture Microdissection

All FFPE human brain samples were purchased from the Netherlands Brain Bank (Amsterdam/The Netherlands) under the regulatory conditions of the Boehringer Ingelheim corporate policy regarding the acquisition and use of human biospecimen. Brains were sectioned at 10 μm thickness using a microtome, collected on polyethylene naphthalate membrane glass slides (Carl Zeiss Microscopy GmbH, Jena, Germany) and air dried over night at room temperature. Prior to LCM, sections on glass slides were deparaffinized using two washes of xylene (3 min each) followed by ethanol (100%, 96%, 70%) for 1 min each. Laser microdissection and pressure catapulting of ∼3,000 substantia nigra cells was performed using a 10 × objective under brightfield optics on a PALM MicroBeam system (Carl Zeiss Microscopy GmbH). The area equivalent to ∼3000 cells was estimated by counting and averaging the cell number of three squares from tissue sections stained with hematoxylin. An amount of ∼3000 cells reflected an area of 1–3 mm^2^ sample depending. Isolated cells were collected in an adhesive cap (Carl Zeiss Microscopy GmbH) and resuspended in extraction buffer followed by the preparation steps described below (see step-by-step protocol in supplemental data for details).

##### Protein Extraction

Unstained FFPE human substantia nigra sections, which were analyzed without LCM (*e.g.* for comparison of buffers), were sectioned using a microtome (4 μm thick), transferred into 1.5 ml tubes and deparaffinized by incubation with 500 μl heptane for 1 h. After the addition of 100 μl methanol samples were vortexed thoroughly and centrifuged for 2 min (15,000 × *g*). The supernatant was removed and tissue samples were air-dried followed by resuspension in 100 μl SDS (2% SDS in 300 mm Tris-HCl pH 8.0), sodium deoxycholate (SDC; 1% SDC in 300 mm Tris-HCl pH 8.5) or Rapigest (0.2% Rapigest in 50 mm ammonium bicarbonate (ABC)) extraction buffer. Samples were boiled (25 min, 99 °C, 350 rpm) and sonicated for 20 cycles (Bioruptor^®^ Pico bath sonicator, Diagenode, Belgium; 30 s on, 30 s off) followed by heating for 2 h at 80 °C (500 rpm) and another sonication step (20 cycles). After centrifugation (10 min, 21,300 × *g*) the supernatant was transferred into a new tube. Optionally, the extraction steps were repeated with the remaining tissue. Reversibly oxidized cysteines were reduced with 10 mm DTT (45 min, 50 °C, 1000 rpm) followed by alkylation of free thiols with 20 mm iodoacetamide (45 min, 22 °C, 1000 rpm, in the dark). Proteins were quantified using the fluorometric EZQ^TM^ assay (Thermo Fisher Scientific, Bremen, Germany).

Laser-capture microdissected tissue samples were resuspended in 100 μl SDS extraction buffer followed by the preparation steps described above. The extraction steps were performed twice and both protein extracts were combined.

##### Protein Digestion Using the SP3 Method

Protein extracts in SDS buffer were cleaned and digested with the SP3 method as described previously with modifications (15, 16). Briefly, 10 μl of a 20 μg/μl SP3 bead stock (Sera-Mag SpeedBead carboxylate-modified magnetic particles; GE Healthcare Life Sciences, Freiburg, Germany) for LCM samples (or a 1:10 protein:bead ratio for sections) and 500 μl acetonitrile (ACN; final concentration of 70%) were added to 200 μl of protein extract and incubated for 10 min (1000 rpm). Tubes were mounted on a magnetic rack; supernatants were removed and beads were washed twice with 70% ethanol and once with ACN (1 ml each). Beads were resuspended in 80 μl 50 mm ABC and digested overnight with trypsin (1:50 trypsin:protein ratio for sections, 0.5 μg for LCM samples; Pierce^TM^ trypsin protease, #90058, Thermo Scientific; 37 °C, 1000 rpm). The next day another portion of trypsin (1:50 trypsin:protein ratio for sections, 0.5 μg for LCM samples) was added and further digested for 4 h. Peptides were cleaned by addition of 9 μl 10% formic acid (final concentration of 1%) and 1750 μl ACN (final concentration of 95%) followed by incubation for 10 min. After spinning down (1000 × *g*) tubes were mounted on a magnetic rack and beads were washed once with 1.5 ml ACN. Peptides were eluted from the beads with 50 μl 2% DMSO and acidified with 2.6 μl 20% formic acid (final concentration of 1%) followed by centrifugation (15,000 × *g*). Peptide amounts were quantified using the fluorometric CBQCA assay (Thermo Scientific).

##### In-solution Digestion

Prior to digestion protein extracts in Rapigest buffer were diluted 1:2 with 50 mm ABC. Rapigest and SDC extracts were digested overnight (1:50 trypsin/protein ratio; 37 °C). The next day another portion of trypsin was added (4 h; 1:50 trypsin/protein ratio). followed by acidification to a final concentration of 1% formic acid. After centrifugation (10 min, 21,300 × *g*) samples were desalted by solid-phase extraction using Sep-Pak tC18 cartridges (50 mg; Waters, Eschborn, Germany) according to the manufacturer's instructions. Briefly, cartridges were activated with ACN and equilibrated with 0.1% TFA in water (1 ml each). Samples were loaded, washed five times with 1 ml 0.1% TFA in water and peptides were eluted with 70% ACN/0.1% TFA (1 ml) and dried *in vacuo* in a Concentrator plus (Eppendorf, Hamburg, Germany).

##### TMT Labeling

The required volume for 1.5 μg peptides per sample were dried *in vacuo* in a Concentrator plus and resuspended in 50 μl 100 mm HEPES pH 8.5. TMT10plex tags (Thermo Scientific) were dissolved in anhydrous ACN and added to the peptide sample in a 1:10 peptide/TMT ratio. Additional anhydrous ACN was added to a final volume of 22 μl. Samples were incubated for 2 h (22 °C, 750 rpm). Labeling efficiency was evaluated by desalting aliquots from 20% of the samples with C18 stage tips followed by LC-MS analysis and database search using TMT as variable modification (see supplemental data for details). In case of insufficient labeling more TMT was added. Unreacted TMT was quenched by incubation with 5 μl 5% hydroxylamine for 30 min. Samples belonging to the same batch were combined and dried *in vacuo*.

##### High pH Reversed Phase Fractionation

TMT labeled samples were fractionated using off-line high pH reversed phase chromatography. Dried samples were resuspended in 5% formic acid and loaded onto a 4.6 × 250 mm XBridge BEH130 C18 column (3.5 μm, 130 Å; Waters). Samples were separated on a Dionex Ultimate 3000 HPLC system (Thermo Scientific) with a flow rate of 1 ml/min. Solvents used were water (A), ACN (B) and 100 mm ammonium formate pH 9 (C). While solvent C was kept constant at 10%, solvent B started at 5% for 3 min, increased to 21.5% in 2 min, 48.8% in 11 min and 90% in 1 min, was kept at 90% for further 5 min followed by returning to starting conditions and re-equilibration for 8 min. Peptides were separated into 48 fractions, which were concatenated into 24 fractions and subsequently dried *in vacuo*. Peptides were redissolved in 5% formic acid and analyzed by LC-MS.

##### LC-MS Analysis

Label-free peptides were analyzed on a Q-Exactive Plus mass spectrometer coupled to a Dionex RSLCnano HPLC (Thermo Scientific). Samples were loaded onto a 100 μm × 2 cm Acclaim PepMap-C18 trap column (5 μm, 100 Å) for 6 min with 2% ACN/0.1% formic acid and a constant flow of 4 μl/min. Peptides were separated on a 75 μm × 50 cm EASY-Spray C18 column (2 μm, 100 Å; Thermo Scientific) at 40 °C over a linear gradient from 10% to 35% B in 110 min with a flow rate of 200 nL/min. Solvents used were 0.1% formic acid (A) and 80% ACN/0.1% formic acid (B). The spray was initiated by applying 2.5 kV to the EASY-Spray emitter. The ion transfer capillary temperature was set to 250 °C and the radio frequency of the S-lens to 50%. Data were acquired under the control of Xcalibur software in a data-dependent mode using the top 15 most abundant precursor ions. Full scan MS spectra were acquired in profile mode with a resolution of 70,000 covering a mass range of *m/z* 350–1,400. The automatic gain control (AGC) target was set to 1 × 10^6^ ions with a maximum fill time of 20 ms. Precursor ions were isolated in the quadrupole with a window of *m/z* 1.5 and fragmented in the higher energy collisional dissociation (HCD) cell with a normalized collision energy of 27. Spectra of fragment ions were acquired with a resolution of 17,500, AGC target of 1 × 10^5^ ions and a maximum fill time of 60 ms. Only peptides with a charge between 2 and 6 were considered for fragmentation. Dynamic exclusion of already fragmented precursor ions was 40 ms and peptide match was set to “preferred”.

TMT labeled samples were analyzed on an Orbitrap Fusion Tribrid mass spectrometer coupled to a Dionex RSLCnano HPLC (Thermo Scientific). LC and spray parameters used were the same as described above. Peptides were separated over a linear gradient from 10% to 40% B in 210 min with a flow rate of 200 nL/min. The full scan was acquired in the orbitrap covering the mass range of *m/z* 350 to 1,400 with a mass resolution of 120,000, an AGC target of 2 × 10^5^ ions and a maximum injection time of 50 ms. Only precursor ions with charges between 2 and 7 were selected, with an isolation window of *m/z* 1.6 for fragmentation using collision-induced dissociation in the ion trap with 35% collision energy. The ion trap scan rate was set to “rapid.” The AGC target was set to 4 × 10^3^ ions with a maximum injection time of 180 ms and a dynamic exclusion of 80 s. During the MS3 analysis, for more accurate TMT quantification, 5 fragment ions were co-isolated using synchronous precursor selection in a window of *m/z* 2 and further fragmented with a HCD collision energy of 55%. The fragments were then analyzed in the orbitrap with a resolution of 50,000. The AGC target was set to 5 × 10^4^ ions and the maximum injection time was 115 ms. The cycle duration was 2 s.

TMT batch 4 was measured with the following changes of parameters. Peptides were separated over a linear gradient from 10% to 40% B in 150 min with a flow rate of 200 nL/min. The full scan was acquired with an AGC target of 4 × 10^5^ ions. Precursor ions were isolated with an isolation window of *m/z* 1.2. The ion trap scan rate was set to “turbo”. The AGC target was set to 1 × 10^4^ ions with a maximum injection time of 50 ms and a dynamic exclusion of 60 s. During the MS3 analysis the maximum injection time was set to 86 ms. The cycle duration was 3 s.

##### Data Analysis

Acquired tandem mass spectra were searched against the Uniprot human database (downloaded on 14^th^ of June 2018, 20,349 proteins) using MaxQuant (version 1.6.2.3). Trypsin/P was specified as a cleavage enzyme, allowing up to two missed cleavages and a mass tolerance of 4.5 ppm for precursor ions and 0.5 Da (Ion trap, used for SPS-MS3 scans) or 20 ppm (Orbitrap) for MS2 fragment ions. Carbamidomethylation of cysteine was used as a fixed modification, with oxidation of methionine, deamidation of asparagine and glutamine and acetylation of the protein N terminus used as variable modifications. For a separate search formylation on lysine and methylation on lysine and arginine were included as variable modifications. For TMT data the type of search was set to reporter ion MS3 using TMT10plex. The fractions of all five TMT batches were searched together. For label-free quantification the “match between runs” option in MaxQuant was only applied to the search for the comparison of microdissected samples with intact tissue sections. All other searches of label-free data, regarding the optimization of protein extraction, were performed without this option. A false discovery rate of 1% was applied to peptide and protein identifications. Contaminants and reverse hits were excluded from the results prior to further analysis. All protein intensities were median-normalized to address differences in sample loadings. For TMT data an additional normalization step was applied to address the variation between batches. The sum of median-normalized and isotope-corrected reporter intensities of all ten TMT channels was calculated per batch and protein. The average of sums higher than zero from five batches was calculated. A normalization factor for each batch and protein group was calculated by division of the sum through the average value. Only protein groups with minimum two peptides per protein and minimum five reporter intensity values in 50 samples were considered for further analysis. Also, as shown by correlation analysis (supplemental Fig. S9), replicate 7.1 was excluded as an outlier.

Hierarchical clusters, heat maps, correlation matrices, box plots and volcano plots were created using Instant Clue (version 0.5.3) (17) and Venn diagrams were created in DataShop (version 1.2; https://peptracker.com/ds/). The histogram was generated with Perseus (version 1.6.2.3) (18), whereas other plots were prepared with Excel. Functional enrichment and pathway analysis was performed with the DAVID tool (19, 20).

##### Experimental Design and Statistical Rationale

To test the three different extraction buffers, four technical replicates were analyzed per buffer using the same donor from substantia nigra. For the comparison of microdissected substantia nigra with intact tissue sections 11 replicate samples from intact sections and 13 replicates from microdissected samples were analyzed, which were prepared from five different donors. An unpaired, two-tailed Student's *t* test (equal variance) was performed to identify proteins with significant higher or lower intensities (*p* ≤ 0.01 and *p* ≤ 0.05). In total, 46 replicate samples of 15 healthy donors from microdissected substantia nigra were analyzed in five separate TMT batches. Additionally, four mixes of samples were analyzed in batches 3–5. An overview of the distribution of replicate samples in batches is shown in supplemental Tables S1 and S2.

## RESULTS

### 

#### 

##### Protein Extraction from FFPE Tissue

**Fig. 1.**
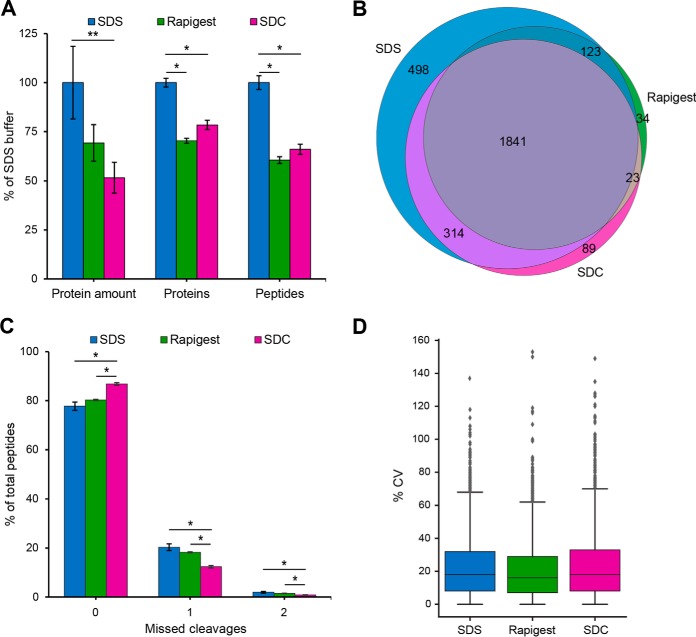
**Optimization of protein extraction from FFPE tissue samples.** *A*, Protein amount extracted from FFPE human substantia nigra (1 section, 4 μm) and number of protein groups (minimum 2 peptides per protein) and unique peptides identified using different protein extraction buffers. Data is shown as means ± S.D. (*n* = 4, * *p* ≤ 0.001, ** *p* ≤ 0.02) and as % of SDS buffer (100% = 45 μg protein, 2370 protein groups, 18,560 peptides). *B*, Venn diagram representing the number of identified protein groups, which are unique and common in samples from different extraction buffers. *C*, Fraction of peptides with zero, one and two missed cleavages. Data is shown as means ± S.D. (*n* = 4, * *p* ≤ 0.001). *D*, Box Plot showing the coefficient of variation (CV) of median-normalized protein intensities from technical replicates (*n* = 4).

Formalin fixation leads to the cross-linking of formaldehyde with several amino acid side chains, including lysine, arginine, histidine and cysteine. This preserves the tissue structure, but also results in poor protein solubilization from FFPE tissue. Therefore, heating of FFPE tissue samples is commonly used to reverse cross-linking with formaldehyde (2). Here, three different protein extraction buffers, containing either 2% SDS, 1% SDC or 0.2% Rapigest, were compared to optimize the amount of extracted protein and the number of protein/peptide identifications. A higher protein amount (>30%) was extracted from tissue using the SDS buffer, compared with the Rapigest and SDC buffers (Fig. 1*A*). Also, significantly (*p* ≤ 0.001) more protein groups (>20%) and unique peptides (>30%), were identified using SDS buffer.

A Venn diagram comparing proteins identified with the different extraction buffers tested reveals that 63% of proteins were identical in all samples (Fig. 1*B*). Additionally, ∼11% were present in samples from the SDS and SDC buffer extracts. Nearly 17% of all identified proteins were only present in samples from the SDS extraction buffer. The number of missed cleavages in SDS (22%) and Rapigest (20%) samples was significantly (*p* ≤ 0.001) higher than in those using SDC buffer (13%) (Fig. 1*C*). The coefficient of variation from four technical replicates, ranging from 16 to 18%, was similar for all three buffers (Fig. 1*D*).

Overall, the SDS containing buffer showed the best results and was consequently chosen for use in the optimized protocol. Additionally, we tested altering the concentration of SDS to investigate if the extracted protein amount could be further increased. However, comparison of buffers with 2 and 5% SDS, respectively, showed little or no difference in either the resulting peptide recovery, or in the numbers of protein/peptide identifications (supplemental Fig. S1*A*). Eighty-five percent of the identified proteins were identical in samples from both buffers (supplemental Fig. S1*B*).

After initial protein extraction, some residual tissue often remains. Thus, an additional extraction step was performed to determine if this could improve total protein recovery. On average, up to an additional ∼15% of the protein amount from the first extract could be obtained with a second extraction. With an equal peptide amount injected, 14% less proteins and 20% less peptides were identified from the second extraction (supplemental Fig. S2*A*). Furthermore, most of the identified proteins were already detected from the first extract (supplemental Fig. S2*B*). However, most protein intensities were lower compared with those from the first extract (supplemental Fig. S2*C*). Thus, a second solubilization step typically does not extract a significant number of additional new proteins from the tissue, but rather increases the total levels of the previously extracted proteins.

To identify specific cells of interest in a morphological context, staining of the tissue sample is often required. Therefore, the influence of IHC and H&E staining of proteins in FFPE tissues was investigated. Sections were stained with rabbit anti-tyrosine hydroxylase pAb using diaminobenzidine and consequently the impact of IHC on proteins was investigated. As shown in supplemental Fig. S3*A*, there was no significant difference in protein and peptide identifications between stained and unstained samples. Also, the fraction of missed cleavages was similar (supplemental Fig. S3*B*). The correlation matrix in supplemental Fig. S3*C* shows clustering of samples based on the donor, instead of staining. Overall, the data show that immunohistochemical staining with diaminobenzidine has little or no negative impact on the efficiency of extraction and detection of tissue proteins.

In comparison, H&E staining resulted in a negative impact on the obtained peptide amount, compared with nuclear- and unstained tissue with a loss of up to 80% (supplemental Fig. S4*A*). However, protein and peptide identifications remained similar, when an equal peptide amount was injected (supplemental Fig. S4*B* and S4*C*). The sample preparation was modified to improve the obtained peptide amount and reduction and alkylation were performed after the SP3 protein wash step, followed by tryptic digestion. Using this modified protocol, the peptide recovery from H&E stained tissue was similar to the one from unstained tissue. The correlation matrices in supplemental Fig. S4*D* and S4*E* confirm these data. Samples prepared with the usual protocol (reduction/alkylation before SP3 protein clean up) clustered on the staining patterns. When using the modified protocol (reduction/alkylation after SP3 protein clean up), samples clustered by donor identity.

##### SP3 Digest

As SDS will interfere with protein digestion and MS measurements, samples need to be cleaned to remove SDS before tryptic digestion. This was done using the SP3 digest method. However, when using the original method, significant sample loss occurred. Thus, protein and peptide washing steps were optimized. In the protein wash step, different pH values of the washing solution (from pH 3 to pH 8) were tested. Additionally, the final concentration of ACN was increased to 70%. Twenty percent more proteins could be recovered, using a solution with pH 8 and 70% ACN, compared with the original conditions with pH 3 and 50% ACN (supplemental Fig. S5*A*). Furthermore, we tested whether the peptide wash step after digestion can be removed, considering that the digestion solutions ammonium bicarbonate and triethylammonium bicarbonate can easily be evaporated. However, it was shown in label-free MS measurements from samples after digestion that the protein wash was not enough to remove SDS completely. SDS contamination present in the sample interfered with the analysis, as shown in supplemental Fig. S5*C*. In comparison, a sample prepared with the peptide wash step after digestion showed a homogenous elution of peptides over the whole retention time range (supplemental Fig. S5*D*). Therefore, different washing approaches were tested, *e.g.* the use of a sonication bath, shaker, rotator and pipetting. However, they did not improve the outcome, showing that the peptide wash after digestion is required to remove any residual SDS present in the sample. Next, therefore, we optimized this step to overcome low peptide recovery seen using the original conditions, *i.e.* with 95% ACN in the washing solution. Acidification to a final concentration of 1% formic acid, before addition of ACN to a final concentration of 95%, improved the recovery of peptides by 60% (supplemental Fig. S5*B*). Overall, with the described modifications to the original protocol, the recoveries of proteins and peptides were improved significantly for both washing steps.

##### Enrichment of Neuron-specific Proteins in Microdissected Samples Compared with Intact Tissue Sections

Laser-capture microdissection enables the purification of cell subpopulations from heterogeneous tissue specimens. Therefore, even minor cell subpopulations can be enriched using this technique. Here, we compared microdissected samples from substantia nigra tissue specimens with intact sections to test the efficiency of LCM (supplemental Fig. S6). For each of five healthy donors, intact tissue sections and 3000 cells, isolated with LCM, were analyzed using a label-free shotgun MS approach.

**Fig. 2.**
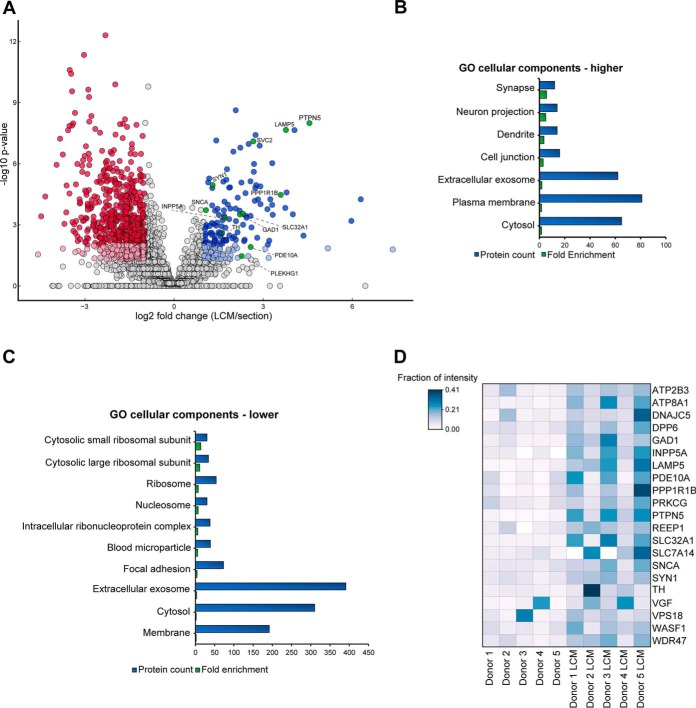
**Comparison of microdissected tissue samples with intact sections.** *A*, Volcano Plot representing the log2 fold changes in protein intensities *versus* the negative log10-transformed *p* values (two-tailed, equal variance *t* test) of microdissected substantia nigra (∼3000 cells) *versus* intact tissue sections (10 μm). Data is based on 11 replicate samples from intact sections and 13 replicates from microdissected samples, which were prepared from total 5 biological replicates. Proteins with fold changes ≥2 (blue) or ≤2 (red) and *p* values ≤0.01 (dark) or ≤0.05 (light) are displayed in color. Neuron-specific proteins are presented in green. *B*, GO cellular components enriched in microdissected substantia nigra compared with intact sections (Benjamini-corrected *p* value <0.01, protein count ≥10). The human proteome was used as background. *C*, GO cellular components with lower intensity in microdissected substantia nigra compared with intact sections (Benjamini- corrected *p* value <0.01, protein count ≥10). The human proteome was used as background. *D*, Heat map representing the fraction of intensity of microdissected samples and intact sections for neuron-specific proteins.

The volcano plot in Fig. 2*A* shows a high number of proteins with a lower intensity in microdissected samples, compared with sections. However, 182 proteins showed a minimum 2-fold increase in protein intensity. Bioinformatic analysis of these proteins compared with the human proteome revealed an enrichment of neuron-specific GO cellular components, including synapse, neuron projection and dendrite (Fig. 2*B*). Conversely, 736 proteins had a minimum 2-fold lower intensity compared with entire sections. These were enriched for the GO terms ribosome and nucleosome, as well as the terms extracellular exosome, membrane and cytosol (Fig. 2*C*). When using a smaller and brain-specific protein set as background (identified proteins from intact sections and microdissected substantia nigra) the enriched GO cellular components of proteins with lower intensity did not change (supplemental Fig. S7*B*). However, proteins with higher intensity in microdissected tissue samples showed only enrichment of two GO terms, which did not include any neuron-specific ones (supplemental Fig. S7*A*).

As shown in Fig. 2*D*, neuron-specific proteins were detected with higher intensity in microdissected samples from most of the donors, compared with the corresponding tissue sections. For example, tyrosine hydroxylase, the marker for dopaminergic neurons, which are present in substantia nigra tissue, was enriched ∼8-fold in donor 2. Alpha-synuclein (SNCA), a component of Lewy bodies, which are a pathological hallmark of Parkinson's disease, was 2.5-fold higher in donors 3 and 5. Tyrosine-protein phosphatase non-receptor type 5 (PTPN5), a brain-enriched protein involved in the regulation of synaptic plasticity, was significantly enriched in all donors, with a minimum 8-fold higher intensity in microdissected samples. Protein phosphatase 1 regulatory subunit 1B (PPP1R1B), a dopamine- and cAMP-regulated neuronal phosphoprotein, showed an average increase of ∼12-fold. Overall, the data show the efficacy of LCM for the enrichment of not only specific cell subpopulations, but also proteins, which are only expressed in these subpopulations and are likely to be suppressed by higher abundance proteins from the surrounding proteome.

##### Analysis of the Proteome of Substantia Nigra

The optimized protocol was applied to laser-capture microdissected human substantia nigra tissue. In total, 46 replicate samples from 15 healthy donors were analyzed in five TMT batches. The distribution of donors and technical replicates across the batches is shown in supplemental Tables S1 and S2. As part of the ongoing optimization process, there were variations in the method used between the separate TMT batches. Specifically, 16 fractions were analyzed in batches 1 and 2, resulting in the identification of >4300 protein groups (minimum two peptides per protein) per batch. Next, the number of fractions was increased to 24, resulting in ∼4800 protein group identifications in batch 3 (Table I). Shorter gradient and run times were tested in batch 4, which reduced the time required to analyze 24 fractions but also resulted in significantly lower protein identifications. Thus, for batch 5, the previous 4 h MS method was used. Compared with label-free shotgun analysis of the same samples it was possible to increase the number of quantified protein groups and unique peptides by minimum 220% using 16 fractions and even by minimum 300% when analyzing 24 fractions (supplemental Fig. S8).

**Table I:** Overview of protein and peptide identifications in all analyzed TMT batches. *Minimum 2 peptides per protein

Batch	Fractions	# Protein groups (2*)	# Unique peptides
1	16	4343	29,887
2	16	4424	31,941
3	24	4812	35,725
4	24	3514	21,260
5	24	3606	21,279
Total	104	5677	53,475

Overall, 5677 protein groups (with minimum two peptides per protein) and 53,475 unique peptides were identified (Table I, supplemental Tables S3, S4, S5). Per replicate sample 3500–4800 protein groups and 21,000–35,000 unique peptides were quantified. When including formylation and methylation as variable modifications around 200 protein groups and 1,400 unique peptides per batch were identified additionally (supplemental Table S6). 4.7% and 3.3% of identified peptides were formylated or methylated.

When analyzing multiple TMT batches, the effect of batch variation, as shown in supplemental Fig. S9*A*, must be controlled for. The correlation of protein intensities for samples within batches is high, with typical Pearson coefficients above 0.9, whereas samples from different batches show a lower correlation, with coefficients of ∼0.8. Therefore, a normalization strategy was applied to the data, which included median-normalization, addressing differences in sample loadings, as well as batch normalization, addressing the differences between batches. Supplemental Fig. S9*B* shows that the correlation of normalized protein intensities of samples from different batches increased to >0.9. Sample 7.1 was deemed an outlier and consequently excluded from further analysis. The intensities from technical replicates per donor were averaged. The correlation of protein intensities between donors was high, with Pearson coefficients of >0.95 (supplemental Fig. S10*A*). The technical coefficient of variation from 4–7 technical replicates was between 11 and 16% for donors 1 to 6 (supplemental Fig. S10*B*). The biological coefficient of variation from 15 different donors was 22% (supplemental Fig. S10*C*).

##### Functional Enrichment of the Nigral Proteome

**Fig. 3.**
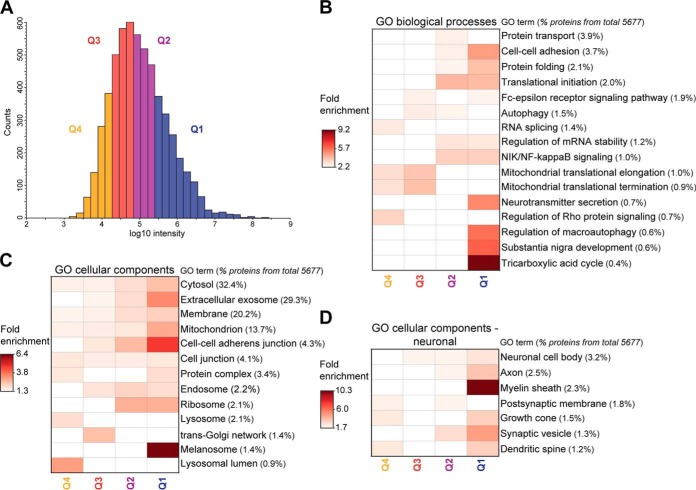
**Functional enrichment of the nigral proteome.** *A*, Histogram representing the intensity range of the nigral proteins identified. Depending on their intensity proteins were divided into four quartiles. *B*, Heat map showing GO biological processes, which were enriched in each of the quartiles (Benjamini-corrected *p* value <0.05, protein count ≥1%). *C*, Heat map showing GO cellular components enriched in each of the quartiles (Benjamini-corrected *p* value <0.05, protein count ≥1%). *D*, Heat map showing neuronal GO cellular components enriched in each of the quartiles (Benjamini-corrected *p* value <0.05, protein count ≥1%).

Nigral proteins were sorted based on their intensity (average of all donors) and divided into quartiles (Fig. 3*A*). Proteins from each quartile were associated with GO cellular components and biological processes, along with Kegg pathways, using DAVID analysis. Regarding GO biological processes proteins participating in RNA splicing, mitochondrial translation and autophagy were enriched in quartiles 3 and 4, whereas TCA cycle, substantia nigra development and neurotransmitter secretion were enriched in quartile 1, corresponding to the highest abundance proteins (Fig. 3*B*). Proteins from the extracellular exosome and proteins annotated with several neuron-specific GO cellular components, such as myelin sheath, synapse, neuron projection, dendrite, axon and synaptic vesicle, were also enriched in quartile 1 (Fig. 3*C* and 3*D*). Lower abundance proteins, from the lysosome and trans-Golgi network, were enriched in quartiles 3 and 4. Annotation of Kegg pathways revealed the enrichment of proteins from the spliceosome and phosphatidylinositol signaling system among the lower abundance proteins, whereas synaptic vesicle cycle, dopaminergic synapse, TCA cycle and oxidative phosphorylation, were among the enriched pathways in quartile 1 (supplemental Fig. S11).

## DISCUSSION

To better understand disease phenotypes it is necessary to analyze specific cell subpopulations, rather than heterogeneous mixtures of cells, which may be present in tissues in varying numbers. The laser-capture microdissection (LCM) technique can be applied to purify either specific cell populations, or tissue regions, for subsequent proteomic analysis. In this study, we optimized a protocol for the proteomic analysis of laser-capture microdissected FFPE tissue, using limited sample amounts. We tested different buffers to achieve efficient protein extraction from FFPE tissue samples, obtaining the best results with SDS containing buffer. SDS was removed from protein extracts, prior to digestion, using a modified SP3 method that improved protein and peptide recoveries. Also, we compared microdissected samples from human substantia nigra with intact tissue sections to investigate the efficiency of LCM for the isolation of distinct cell subpopulations in a morphological context. This resulted in the enrichment of neuron-specific proteins in microdissected samples. Finally, the optimized protocol was applied to analyze a set of FFPE human substantia nigra samples microdissected from healthy donors. Based on the intensity of nigral proteins identified, this demonstrated the enrichment of specific GO terms and Kegg pathways for different protein abundance classes.

Protein extraction of FFPE tissue is challenging, mainly because of the cross-links and modifications induced by formaldehyde treatment. However, heating of FFPE samples is known to reverse these cross-links, resulting in improved protein extraction. Also, different buffer components, including detergents and organic solvents, can enhance protein solubilization from FFPE samples. Thus, SDS in combination with Tris (9, 21) and Rapigest with ammonium bicarbonate (22–24), are widely used in extraction buffers. Therefore, we compared these two compositions with a buffer containing SDC and Tris used in-house, which revealed that the SDS containing buffer provided the best results regarding extracted protein amount and numbers of protein/peptide identifications. This agrees with previous studies, which used SDS for efficient extraction from FFPE tissue (8, 9, 21). SDS concentrations of 2% in extraction buffers for FFPE samples are often used (25–27). However, recently proteins were extracted with a buffer containing 5% SDS (28). Thus, we compared both SDS concentrations, which did not show any significant difference. The SDC buffer yielded also higher protein/peptide identification numbers compared with the Rapigest buffer, despite lower extracted protein amounts. We note that the concentrations of the detergents in the tested buffers were different as it was our aim to compare buffers from published studies with an already in-house used one. However, a conclusion regarding the extraction efficiency of these detergents cannot be drawn from the here described results. Higher concentrations of Rapigest and SDC might increase extracted protein amounts and identification numbers. In addition, two digestion methods were applied, which might differ in sample loss and digestion efficiency resulting in varying numbers of missed cleavages and identifications. However, like the detergents a conclusion regarding a better digestion method cannot be drawn from the presented results.

Tissue proteins were extracted in a buffer containing 300 mm Tris, to support the reversal of cross-links. Usually, concentrations up to 100 mm Tris are used, but as Kawashima *et al.* recently described, a higher concentration of Tris increases the amount of extracted proteins (29). Tris, which contains a primary amine, might act either as a scavenger of released formaldehyde, or as a transamination catalyst. After protein extraction residual tissue was usually still present. It has been shown previously that additional extraction steps can improve the overall yield (30, 31). Lai *et al.* reported protein amounts of over 100% of the first extract in a second and up to 70% in a third extraction (30). However, they homogenized samples only in the second extraction step. Additionally, proteins were extracted in ammonium bicarbonate alone resulting in insufficient protein solubilisation. Here, a second extraction increased the protein amount by an average of ∼15% of the first extract and was implemented into the protocol for the analysis of microdissected substantia nigra samples. However, considering the amount of time the extraction procedure requires, we suggest this step might best be included only when insufficient quantities of proteins are recovered after the first extraction.

For the reliable isolation of morphologically distinct cell populations, staining of proteins of interest (IHC), or histological staining (H&E), is often necessary. Here, we tested the impact of staining techniques on protein recovery and yield. In agreement with previous studies, we demonstrated that IHC didn't alter the number of protein identifications, compared with unstained tissue samples (7). Like recent reports, we showed that H&E staining reduces peptide identifications up to 20%, whereas protein numbers were similar between samples with different stainings (22). However, we observed ∼ 80% loss in the peptides obtained after tryptic digestion. Additionally, the correlation matrix showed clustering of samples based on their staining patterns, rather than donor identity. Therefore, to optimize the protocol, the order of reduction/alkylation and protein clean-up was switched, which increased the peptide quantities recovered after digestion and resulted in samples clustering independent of their staining. This improvement is probably related to the absence of eosin, an acidic dye, which binds to basic amino acid side chains of cytosolic proteins. When present in the buffer, eosin might react with iodoacetamide, considering that eosin-5-iodoacetamide, a thiol-reactive fluorophore, was previously used to derivatize proteins (32, 33). This reaction would result in bulky protein modifications, which can interfere with digestion and increase the number of missed cleavages.

SDS is known to inhibit digestion enzymes and interfere with downstream LC-MS analysis and therefore needs to be removed prior to these steps. Therefore, samples prepared in SDS buffer were processed using the SP3 method (15), which includes a protein clean-up step before and a peptide clean-up step after digestion. However, in our hands, using the original protocol, protein and peptide recoveries were lower than reported. We therefore tested modifications to improve the SP3 protocol. Following previous studies, the protein wash solution was changed to conditions with pH 8 and 70% ACN (34, 35). To avoid sample losses through unnecessary preparation steps and considering that digestion and TMT labeling can be performed in the same buffer system, we tried to remove the peptide wash step. However, chromatograms of samples prepared without clean-up showed interference, probably caused by the presence of SDS suppressing peptide ionization. Conversely, samples prepared using the peptide wash step did not show any interference with contaminants. Therefore, the peptide wash step was retained and supplemented by acidification of the washing solution to improve peptide recovery. We believe this improves the efficiency of both the original protocol and subsequent studies using the SP3 method.

The comparison of analyzing microdissected samples and intact sections showed a higher number of protein identifications in the intact tissue sections, with the majority having a higher intensity. This contrasts with a recent study, where more proteins were identified in microdissected samples, compared with intact sections from breast cancer tissues (36). Neuron-specific proteins were enriched in microdissected samples, whereas ribosomal and nucleosomal proteins demonstrated lower intensities. The substantia nigra consists of different cell populations, including different neurons (dopaminergic, GABAergic, glutamatergic) and glial cells (astrocytes, oligodendroglia and microglia) (37, 38). Reports suggest that glial cells are present in a higher number compared with dopaminergic neurons (39). Here, we microdissected 3000 cells from areas containing dense dopaminergic neurons, which are visible by brown-pigmented neuromelanin. Thus, the results demonstrate that, by using LCM, we could enrich the neuronal cells compared with the surrounding tissue, which contains more glial cells.

Enriched GO terms from proteins with lower intensity in microdissected samples didn't differ when using different protein sets (human and brain-specific proteome) as background for the functional enrichment analysis. However, neuron-specific GO terms from proteins with higher intensity were only enriched, when the human proteome was used as background showing that there is no difference between both areas analyzed regarding the identity of neuronal proteins. Nevertheless, several neuron-specific proteins were detected with a significantly higher intensity in microdissected samples including tyrosine hydroxylase, a marker for dopaminergic neurons (40). Alpha-synuclein (SNCA) is involved in Parkinson's disease via the accumulation and formation of Lewy bodies, which are a pathological hallmark of Parkinson's disease (41). In the substantia nigra SNCA is expressed in neuronal cell bodies and synapses as well as TH-positive neurons (42).

This study analyzed a total of five TMT batches of microdissected samples. However, they were not all measured using the exact same procedure, with differences in batch design regarding the number of fractions, number of technical replicates and LC-MS methods, as described in the results. With this limitation, we decided to combine the results of all five batches, despite their differences in analysis and design, to provide a “proof of principle” of laser-capture proteomics, using small sample amounts from a set of healthy donors. When more than one TMT batch is analyzed, the problem of variation between batches occurs, as shown here in the correlation matrix and reported in previous studies (43). Usually, a reference sample is included in all batches, which is then used for normalization (43, 44). Here, we applied an alternative normalization strategy that involved summing up intensities of all ten channels per protein within a batch and using these sums from all batches to calculate a reference value for normalization. Using this strategy, the variation between batches could be reduced. This provides a good alternative for batch normalization in cases where no common reference samples are available.

In this study we used a peptide:TMT ratio of 1:10. The standard ratio suggested from the manufacturer is 1:8. However, the number of overlabeled peptides (average of 13%, data not shown), which have a TMT modification on Ser, Thr or His, didn't change compared with the commonly used ratio (45). Although most formaldehyde cross-links can be reversed by heating, there are still irreversible modifications with formaldehyde present. It has been shown recently that formylation and methylation on lysine are common modifications in FFPE tissue samples (46–48). However, these modifications are rarely included in the database search in studies using FFPE material. Here, we performed an additional search including formylation and methylation as variable modifications. Consequently, the number of identifications increased by approx. 5%.

Recently, a growing number of proteomic studies have been performed using LCM to analyze cellular subpopulations in a pathological context. For instance, Clair *et al.* purified lung alveoli from cryo sections with a total area of 4 mm^2^ and identified more than 3446 proteins (minimum 2 peptides per protein) in a label-free approach (49). Shapiro *et al.* isolated pancreatic acinar cells from FFPE tissue with a total area of 1 mm^2^ (∼10,000 cells corresponding to 2–3 μg protein) and identified in total 2995 proteins (24) in a murine model of caerulein-induced pancreatitis, with a label-free shotgun approach. Buczak *et al.* isolated different tumor sectors and compared tumor with non-tumor tissue of hepatocellular carcinoma from FFPE tissue. 40 mm^2^ per region (5–10 μg peptide) were dissected and 5838 and 5659 protein groups were identified per batch in a TMT-based approach using fractions (9). In the current study we isolated as little as 3000 cells, corresponding to areas of 1–3 mm^2^ and 1–4 μg peptide and identified in total over 5600 protein groups with a range of 3500 to 4800 protein groups per batch. Considering that protein amount and content vary between different tissues and cells, we can conclude that protein numbers from this study agree with, if not higher than, protein detection reported in most of the previous studies. Interestingly, Davis *et al.* reported recently the analysis of Betz and Purkinje cells isolated from frozen human brain tissue in a label-free shotgun approach using only 150 cells, which resulted in the identification of 2718 and 2025 protein groups with minimum 2 peptides per protein. In this study microdissected tissue was directly collected in RIPA buffer followed by digestion with the SP3 method (50). Additionally, it was shown that digestion directly in the cap could significantly improve identification numbers for a low amount of cells (100–400) revealing that the transfer of microdissected tissue from the cap into tubes is a critical point, which could lead to significant sample losses when working with small sample amounts. Therefore, collection of microdissected tissue directly in buffer might further improve results.

**Fig. 4.**
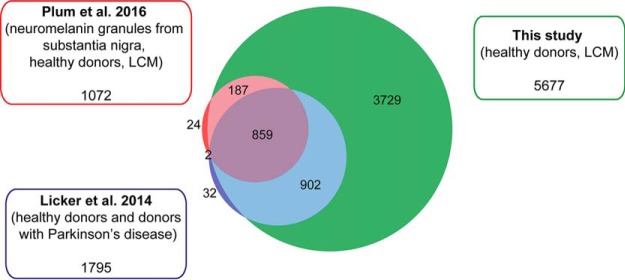
**Comparison of protein identifications between different studies.** Venn diagram showing the number of identified proteins from published studies compared with the present one.

Several proteomic studies have analyzed the substantia nigra, motivated by its importance in the pathology of Parkinson's disease and other neurodegenerative diseases (5, 11–14). However, in previous studies, the region has often been macrodissected with a scalpel, which is not as precise as LCM. Also, these studies were often performed with cryo sections, rather than with the FFPE tissue used here. For example, in 2014 Licker *et al.* analyzed the substantia nigra from healthy donors and donors with Parkinson's disease and identified 1795 proteins (13), whereas Plum *et al.* analyzed the neuromelanin granules, which were isolated by LCM and identified 1072 proteins (5). Ninety-seven percent of these proteins were also detected in the present study (Fig. 4).

We present an optimized protocol for laser-capture proteomics using small sample amounts of FFPE tissue, which was applied to human substantia nigra samples. We demonstrate a detailed investigation of the protein extraction step by comparing different buffers and testing the influence of IHC and H&E staining and show the efficiency of LCM by comparing microdissected samples with intact sections. The substantia nigra, with all its different cell populations, was used for this method development study, rather than a specific cell population, such as tyrosine hydroxylase positive neurons. In conclusion, this study is, to the best of our knowledge, the most detailed proteomic data set of human substantia nigra reported so far. We note that in Parkinson's disease the loss of dopaminergic neurons occurs in the substantia nigra, whereas those in the ventral tegmental area are more resistant to degeneration. It will therefore be important in future to distinguish neurons from both brain regions and analyze their proteomes during Parkinson's disease. The laser-capture proteomic method established here can now be applied to analyze the protein expression of dopaminergic neurons in a pathological context.

## DATA AVAILABILITY

The raw files used for this analysis have been deposited to the ProteomeXchange Consortium (51) via the PRIDE partner repository (52) with the data set identifier PXD016563, along with the full MaxQuant (53) output.

## Supplementary Material

Supplemental data

Supplementary tables S1-3

Supplementary tables S4-5

## Footnotes

^1^ The abbreviations used are:

FFPE: formalin-fixed paraffin-embedded
LCM: laser-capture microdissection
SP3: single-pot solid-phase-enhanced sample preparation
TMT: tandem mass tag
GO: gene ontology
IHC: immunohistochemistry
H&E: haematoxylin & eosin
SDC: sodium deoxycholate
ABC: ammonium bicarbonate
ACN: acetonitrile
AGC: automatic gain control
HCD: higher-energy collisional dissociation
TCA: tricarboxylic acid.
