# Supplementary material for: Quantitative Profiling of the Human Substantia Nigra Proteome from Laser-capture Microdissected FFPE Tissue
Source: Mol Cell Proteomics. 2020 Mar 4;19(5):839–51. doi: 10.1074/mcp.RA119.001889 (PMC7196589; doi:10.1074/mcp.RA119.001889)
Supplement: Supplemental data [file 157418_1_supp_477977_q5y6y9.docx]

**Supplemental data**

**Quantitative profiling of the human substantia nigra proteome from laser-capture microdissected FFPE tissue**

Eva Griesser^1,2^, Hannah Wyatt^2^, Sara Ten Have^1^, Birgit Stierstorfer^2^, Martin Lenter^2^ and Angus I. Lamond^1^

^1^Centre for Gene Regulation and Expression, School of Life Sciences, University of Dundee, Dundee, DD1 5EH United Kingdom, ^2^Drug Discovery Sciences, Boehringer Ingelheim Pharma GmbH & Co. KG, Biberach an der Riss, Germany

Corresponding author: Angus I. Lamond, [a.i.lamond@dundee.ac.uk](mailto:a.i.lamond@dundee.ac.uk)

**Supplemental methods**

**Immunohistochemical staining**

Brains were sectioned at 10 µm thickness using a microtome, collected on polyethylene naphthalate membrane glass slides (Carl Zeiss Microscopy GmbH) and air dried over night at room temperature. Sections were deparaffinised using two washes of xylene (3 min each) followed by rehydration with 100% (2x), 96% (2x) and 70% ethanol (2x) for 1 min each and a short rinse in water. After washes with PBS (2 x 5 min) sections were blocked with 5% goat serum and 2% BSA in PBS for 30 min followed by PBS washes (2 x 2 min). Sections were incubated with rabbit anti-tyrosine hydroxylase primary antibody (1:500; Calbiochem, #657012) in 5% goat serum and 2% BSA in PBS (60 min) followed by biotinylated goat anti-rabbit IgG secondary antibody (1:500; Vector Labs, #BA-1000) in 5% goat serum and 2% BSA in PBS (30 min) with each step followed by PBS washes (2x 5 min). Bound antibodies were detected by incubation with the reagent of the Vectastain® Elite® ABC HRP kit (Vector Labs, #PK-6100) in PBS (30 min) followed by PBS washes (2 x 5 min), incubation with the substrate working solution from the DAB peroxidase (HRP) substrate kit as suggested (Vector Labs, #SK-4100) and PBS washes (3 x 5 min). Finally, sections were counterstained with Mayer’s hemalum solution (1:10 in water), rinsed with tap water (10 min), dehydrated with 96% ethanol (2 x 1 min), 100% ethanol (2 x 1 min) and xylene (2 x 3 min) and air-dried. All incubations and washes were performed at room temperature.

**Haematoxylin & eosin (H&E) staining**

After deparaffinization as described above sections were stained with haematoxylin (Papanicolaou 1b) for 10 min, followed by short dips in water and 1% HCl in ethanol and a water rinse (9 min). After short dips in ethanol (80%, 96%) sections were stained with eosin-phloxine (1.6 mM eosin G and 0.23 mM phloxine B in 85% ethanol with 1.1% acetic acid) for 2 min. Sections were dehydrated with 96% ethanol (2 x 1 min), 100% ethanol (2 x 1 min) and xylene (2 x 3 min) and air-dried.

**Check for TMT labelling efficiency**

Aliquots (approx. 5% of total sample; e.g. 4 µL from 72 µL) were taken after TMT labelling to check for TMT labelling efficiency and diluted with 46 µL 0.1% TFA in water. Stage tips were prepared by punching a 3M^TM^ Empore C18 disk with a HPLC fitting (ID: 1.59 mm, 1/16 inch) and mounting the material into 200 µL tips. A resin slurry of the Poros® 20 R2 reversed-phase resin (Applied Biosciences by Thermo Scientific) in 70% ACN/0.1% TFA using a 1:2 resin:solvent ratio was prepared and 15 µL of the slurry was added to the stage tip. Tips were centrifuged for 1 min (1000 g) to remove the solvent. The resin was activated with 30 µL 70% ACN/0.1%TFA and equilibrated with 30 µL 0.1% TFA. The sample was loaded (50 µL) and the resin was washed three times with 30 µL 0.1% TFA (after each step: centrifugation for 2 min at 1000 g). Peptides were eluted with 50 µL 70% ACN/0.1% TFA and samples were dried *in vacuo* in an Eppendorf concentrator plus. Samples were resuspended in 5% formic acid and analysed on a Q-Exactive Plus mass spectrometer coupled to a Dionex RSLCnano HPLC (Thermo Scientific). Samples were loaded onto a 100 µm × 2 cm Acclaim PepMap-C18 trap column (5 µm, 100 Å) for 6 min with 2% ACN/0.1% formic acid and a constant flow of 4 µL/min. Peptides were separated on a 75 µm × 50 cm EASY-Spray C18 column (2 µm, 100 Å; Thermo Scientific) at 40 ºC over a linear gradient from 10% to 35% B in 80 min with a flow rate of 200 nL/min. Solvents used were 0.1% formic acid (A) and 80% ACN/0.1% formic acid (B). The spray was initiated by applying 2.5 kV to the EASY-Spray emitter. The ion transfer capillary temperature was set to 250 °C and the radio frequency of the S-lens to 50%. Data were acquired under the control of Xcalibur software in a data-dependent mode using the top 15 most abundant precursor ions. Full scan MS spectra were acquired in profile mode with a resolution of 70,000 covering a mass range of *m/z* 350-1,400. The automatic gain control (AGC) target was set to 1x10^6^ ions with a maximum fill time of 20 ms. Precursor ions were isolated in the quadrupole with a window of *m/z* 1.5 and fragmented in the higher energy collisional dissociation (HCD) cell with a stepped normalized collision energy of 30 and 33. Spectra of fragment ions were acquired with a resolution of 17,500, AGC target of 1x10^5^ ions and a maximum fill time of 100 ms. The first mass was set to *m/z* 100. Only peptides with a charge between 2 and 6 were considered for fragmentation. Dynamic exclusion of already fragmented precursor ions was 40 ms and peptide match was set to “preferred”.

Acquired tandem mass spectra were searched against the Uniprot human database (downloaded on 14^th^ of June 2018, 20,349 proteins) using MaxQuant (version 1.6.2.3). Trypsin/P was specified as a cleavage enzyme, allowing up to two missed cleavages and a mass tolerance of 4.5 ppm for precursor ions and 20 ppm (Orbitrap) for MS2 fragment ions. Carbamidomethylation of cysteine was used as a fixed modification, with oxidation of methionine, deamidation of asparagine and glutamine, acetylation of the protein N-terminus and TMT on lysine and the peptide N-terminus used as variable modifications. A false discovery rate of 1% was applied to peptide and protein identifications. Contaminants and reverse hits were excluded from the results prior to further analysis. The labelling efficiency was calculated by dividing the number of TMT-labelled peptides through the number of total peptides for lysine and peptide N-terminus separately.

**Step-by-step protocol**

Laser-capture microdissection:

- Cut tissue sections from paraffin blocks with 10 µm thickness using a microtome
- Mount sections on PEN-membrane glass slides (Carl Zeiss Microscopy GmbH, Jena, Germany, #415190-9041-000) and let them air-dry overnight at room temperature
- Deparaffinise tissue sections by incubating them in xylene for 3 min (2x) followed by sequential rehydration (1 min each; 100% ethanol (2x), 96% ethanol (2x), 70%)
- Let sections air-dry at room temperature
- Optional: immunohistochemical or H&E staining of sections
- Mount glass slides with sections on the stage of a PALM MicroBeam system (Carl Zeiss Microscopy GmbH)
- Use a 10 × objective under brightfield optics for laser microdissection and pressure catapulting (LMPC) of cells using the PALM RoboSoftware 4.3
- Mark cells or area of interest and use the RoboLPC method to microdissect and catapult/capture them into adhesive caps (Carl Zeiss, #415190-9211-000)

Heptane deparaffinization of intact tissue sections:

- *This deparaffinization protocol can be applied to intact tissue sections, which are analysed without staining and laser-capture microdissection and are directly transferred into tubes. Another possibility is the transfer of intact sections from the glass slides into tubes after xylene deparaffinization as described above.*
- Cut tissue sections from paraffin blocks with 4 µm thickness using a microtome
- Transfer sections into 1.5 mL tubes
- Deparaffinise tissue sections by incubation with 500 µL heptane for 1 h
- Add 100 µL methanol and vortex samples thoroughly
- Centrifuge samples for 2 min (15,000 g)
- Remove supernatant and let tissue samples air-dry

Protein extraction:

- Resuspend laser-capture microdissected tissue in 100 µL extraction buffer (2% SDS in 300 mM Tris-HCl pH 8.0). Get microdissected tissue from caps into suspension by turning tubes upside down several times and centrifuging them shortly at low speed. *Centrifugation at highest speed can move the white adhesive material from cap into the tube!*
- *Make sure that tissue from cap and walls is in suspension!*
- *Caps can open during the following heating step! If necessary use tube sealing clips, transfer tissue suspension into the appropriate tubes (e.g. Eppendorf Safe-Lock), or don’t shake samples to avoid sample loss while heating*
- Heat samples for 25 min (99 ºC, 350 rpm) to reverse formaldehyde crosslinks
- Sonicate samples in a bath sonicator (e.g. Bioruptor® Pico, Diagenode, Belgium or QSonica Q700 with microplate horn, Newtown, CT) for 20 cycles (30 s on, 30 s off)
- Spin down samples
- Incubate samples for 2 h at 80 ºC (500 rpm) to reverse formaldehyde crosslinks
- Sonicate samples in a bath sonicator for 20 cycles (30 s on, 30 s off)
- Spin down samples
- Reduce reversibly oxidized cysteines with 10 mM DTT (45 min, 50 ºC, 1000 rpm)
- Alkylate free thiols with 20 mM IAA (45 min, 22 ºC, 1000 rpm, dark)
- Centrifuge samples for 5 min (22 ºC, 16000 g)
- Transfer supernatant into new tube (2 mL)
- *Optional: if residual tissue is visible: repeat the extraction steps with the remaining tissue and combine with the first extract*

Digestion using the SP3 method (**s**ingle-**p**ot **s**olid-**p**hase-enhanced **s**ample **p**reparation):

- 200 µL protein extract in 2 mL tube (use extraction buffer to reach required volume)
- Preparation of a 20 µg/µL SP3 bead stock:
  - Sera-Mag SpeedBead Carboxylate-Modified magnetic particles, hydrophilic (#45152105050250)/hydrophobic (#65152105050250) (GE Healthcare Life Sciences/GE Healthcare Europe GmbH, Germany)
  - Bead stocks are provided in a concentration is 50 µg/µL
  - Take 200 µL (=10 mg) each of hydrophilic and hydrophobic beads (ratio 1:1)
  - Mount tube on magnetic rack (wait for 2 min) and remove supernatant
  - Wash beads with 1 mL water (3x)
  - Resuspend beads in 1 mL water for a f.c. 20 µg/µL
- Add 10 µL SP3 bead stock
- Add 500 µL 100% ACN (f.c. 70%)
- Incubate for 10 min (22 ºC, 1000 rpm)
- Mount tubes on magnetic rack (2 min)
- Remove supernatant
- Wash beads twice with 70% ethanol and once with ACN (1 mL each), while tube is on rack
- Remove tube from magnetic rack
- Redissolve beads in 80 µL 50 mM ABC through shaking (10 min, 1000 rpm)
- Tryptic digestion overnight (0.5 µg trypsin, 37 ºC, 1000 rpm)
- After digestion acidify with 9 µL 10 % formic acid (f.c. 1%)
- Add 1750 µL 100% ACN (f.c. 95%)
- Incubate for 10 min (RT, 1000 rpm)
- Mount tubes on magnetic rack (2 min)
- Remove supernatant
- Wash beads with 1.5 mL ACN, while tube is on rack
- Redissolve beads in 50 µL 2% DMSO
- Incubate for 10 min (RT, 1000 rpm)
- Mount tubes on magnetic rack (2 min)
- Transfer supernatant (= peptides) into new tube (0.5 mL)
- Acidify with 2.6 µL 20% formic acid
- Centrifuge samples (5 min, 16000 g)
- Quantify peptides with the fluorometric CBQCA assay using the peptide digest assay standard (#C6667 and #23295, both Thermo Scientific)

TMT labelling:

- Take the volume corresponding to the same peptide amount of all samples, which should be combined
- Dry samples *in vacuo* (45 °C; e.g. Eppendorf concentrator plus)
- Redissolve peptides in 50 µL 100 mM HEPES pH 8.5
- *A lower volume might be used in case of insufficient labelling, as peptide and TMT concentrations would be increased: redissolve in 14 µL HEPES buffer*
- Check the pH of the peptide solution with a pH indicator paper (*by dipping tip into solution, no need to pipette*)
- Dissolve 800 µg TMT tags in 80 µL anhydrous ACN (f.c. 10 µg/µL), prepare aliquots of 100 µg TMT in 0.5 mL tubes, dry *in vacuo* and store at -20 °C
- Add TMT labels in a 1:10 peptide:TMT ratio
- Add 100% ACN for total 22 µL (f.c. 30%), *lower volume: 6 µL*
- Incubate samples (2 h, 22 ºC, 600 rpm)
- Take aliquots (5% of sample, e.g. 4 µL from 72 µL) for labelling efficiency check (from 20% of samples) and store samples at -20 °C until check revealed sufficient labelling
- Add 45 µL 0.1% TFA in water
- Clean up aliquots with C18 stage tips
- Prepare stage tips by punching a 3M^TM^ Empore C18 disk with a HPLC fitting (ID: 1.59 mm, 1/16 inch) and mounting the material into 200 µL tips
- Centrifuge stage tips for 2 min at 1000 g (check that resin doesn’t get dry) after each of the following steps:
  - Activate resin with 30 µL 70% ACN/0.1%TFA
  - Equilibrate with 30 µL 0.1% TFA
  - Load sample (50 µL)
  - Wash resin three times with 30 µL 0.1% TFA
  - Elute peptides with 50 µL 70% ACN/0.1% TFA
- Dry samples *in vacuo* (45 °C)
- Resuspend samples in 5% formic acid
- Analyse TMT-labelled peptides by LC-MS
- Perform database search (e.g. with MaxQuant) with the usual parameters using additionally TMT on Lys and peptide N-terminus as variable modification
- When labelling is not sufficient (Lys < 99% and N-term < 95%), add another portion of TMT tags and check again TMT labelling efficiency
- When labelling is sufficient (Lys ≥ 99% and N-term ≥ 95%), quench unreacted TMT with 5 µL 5% hydroxylamine (30 min, 22 ºC, 600 rpm)
- Combine TMT-labelled samples in one tube (2 mL)
- Dry samples *in vacuo* (45 ºC)

High pH reversed phase fractionation:

**Used in this manuscript**

- Resuspend TMT-labelled peptides in 210 µL 5% formic acid, centrifuge (5 min, 16000 g) and transfer into vials
- Load 200 µL on a Dionex Ultimate 3000 HPLC system (Thermo Scientific):
  - Column: Waters XBridge Peptide BEH C18, 130Å, 3.5 µm, 4.6 mm × 250 mm
  - Solvents: A – 100% water, B – 100% ACN, C – 100 mM NH_4_ formate, pH 9.0
  - Gradient:

|  | Retention [min] | Flow [ml/min] | %B | %C | %D |
| --- | --- | --- | --- | --- | --- |
| 1 | 0.0 | 1.000 | 5.0 | 10.0 | 0.0 |
| 2 | 3.0 | 1.000 | 5.0 | 10.0 | 0.0 |
| 3 | 5.0 | 1.000 | 21.5 | 10.0 | 0.0 |
| 4 | 16.0 | 1.000 | 48.8 | 10.0 | 0.0 |
| 5 | 17.0 | 1.000 | 90.0 | 10.0 | 0.0 |
| 6 | 22.0 | 1.000 | 90.0 | 10.0 | 0.0 |
| 7 | 23.0 | 1.000 | 5.0 | 10.0 | 0.0 |
| 8 | 30.0 | 1.000 | 5.0 | 10.0 | 0.0 |

- Collect 48 fractions (21 s each, from 4 to 21 min) concatenated into 24 fractions (by RowByRowMeandering)
- Dry fractions *in vacuo*
- Redissolve peptides in 5% formic acid

**Currently used by the authors**

- Resuspend TMT-labelled peptides in 42 µL 5% formic acid, centrifuge (5 min, 16000 g) and transfer into vials
- Load 40 µL on a Dionex Ultimate 3000 HPLC system (Thermo Scientific):
  - Column: Waters XBridge Peptide BEH C18, 130Å, 3.5 µm, 2.1 mm × 150 mm
  - Solvents: A – 100% water, B – 100% ACN, C – 100 mM NH_4_ formate, pH 9.0
  - Gradient:

|  | Retention [min] | Flow [ml/min] | %B | %C | %D |
| --- | --- | --- | --- | --- | --- |
| 1 | 0.0 | 0.200 | 1.0 | 10.0 | 0.0 |
| 2 | 3.0 | 0.200 | 1.0 | 10.0 | 0.0 |
| 3 | 5.0 | 0.200 | 10.0 | 10.0 | 0.0 |
| 4 | 10.0 | 0.200 | 21.5 | 10.0 | 0.0 |
| 5 | 40.0 | 0.200 | 48.8 | 10.0 | 0.0 |
| 6 | 45.0 | 0.200 | 90.0 | 10.0 | 0.0 |
| 7 | 50.0 | 0.200 | 90.0 | 10.0 | 0.0 |
| 8 | 51.0 | 0.200 | 1.0 | 10.0 | 0.0 |
| 9 | 60.0 | 0.200 | 1.0 | 10.0 | 0.0 |

- Collect 72 fractions (37 s each, from 4 to 49 min) concatenated into 24 fractions (by RowByRowMeandering)
- Dry fractions *in vacuo*
- Redissolve peptides in 5% formic acid

**Supplementary tables**

**Table S6:** Overview of protein and peptide identifications in all analysed TMT batches searched with formylation on lysine and methylation on lysine and arginine. *Minimum 2 peptides per protein.

| **Batch** | **Fractions** | **# Protein groups (2*)** | **# Unique peptides** |
| --- | --- | --- | --- |
| 1 | 16 | 4526 | 31249 |
| 2 | 16 | 4603 | 33396 |
| 3 | 24 | 5057 | 37897 |
| 4 | 24 | 3685 | 22510 |
| 5 | 24 | 3829 | 22765 |
| Total | 104 | 6227 | 57057 |

**Supplemental figures**


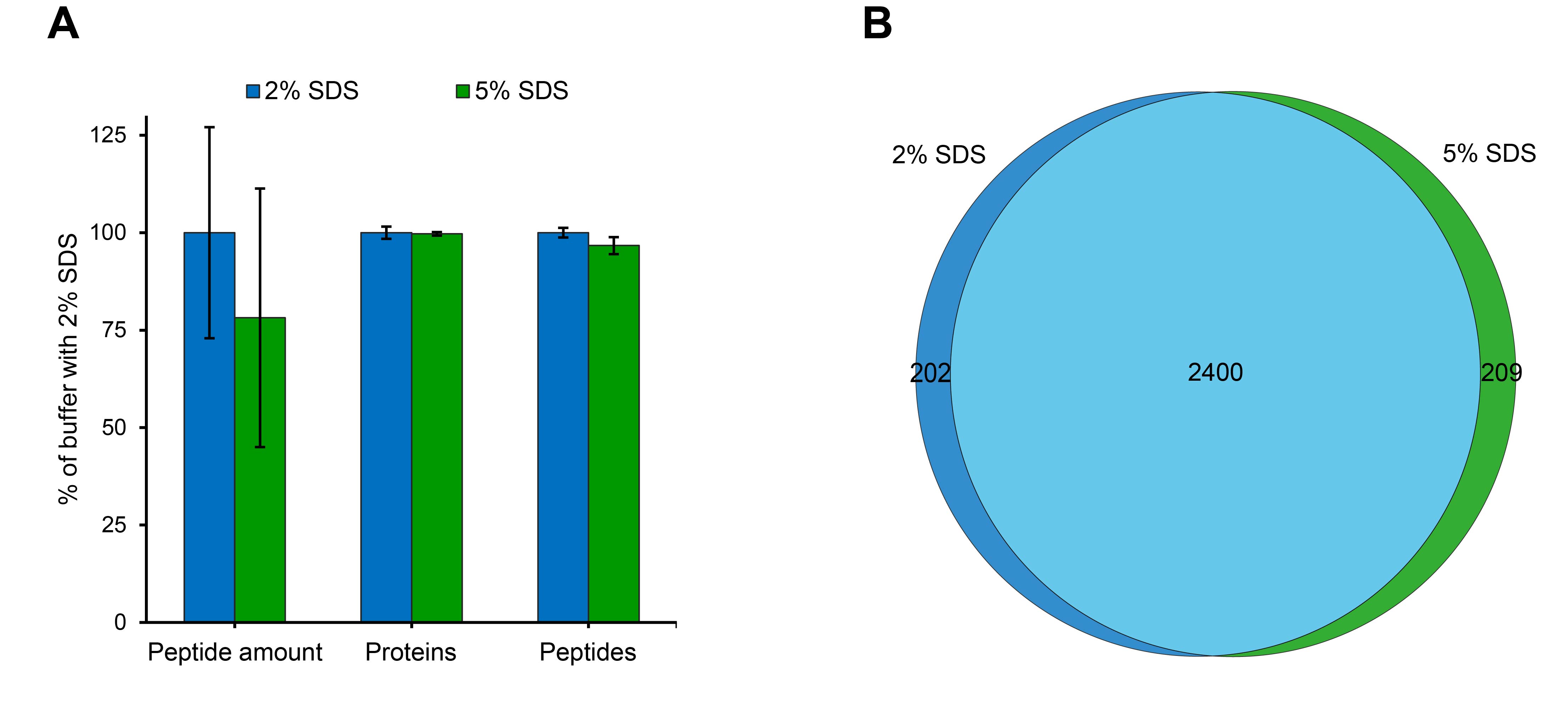


**Figure S1:** Comparison of protein extraction buffers with 2% and 5% SDS. (A) Peptide amount obtained from FFPE human substantia nigra (1 section, 4 µm) and number of protein groups (minimum 2 peptides per protein) and unique peptides identified with extraction buffers containing 2% or 5% SDS. Data is shown as means ± SD (n=3) and as % of 2% SDS (100% = 18.9 µg peptide, 2,230 protein groups, 17,123 peptides). (B) Venn diagram representing the number of identified protein groups, which are unique and common in samples from different extraction buffers.

**
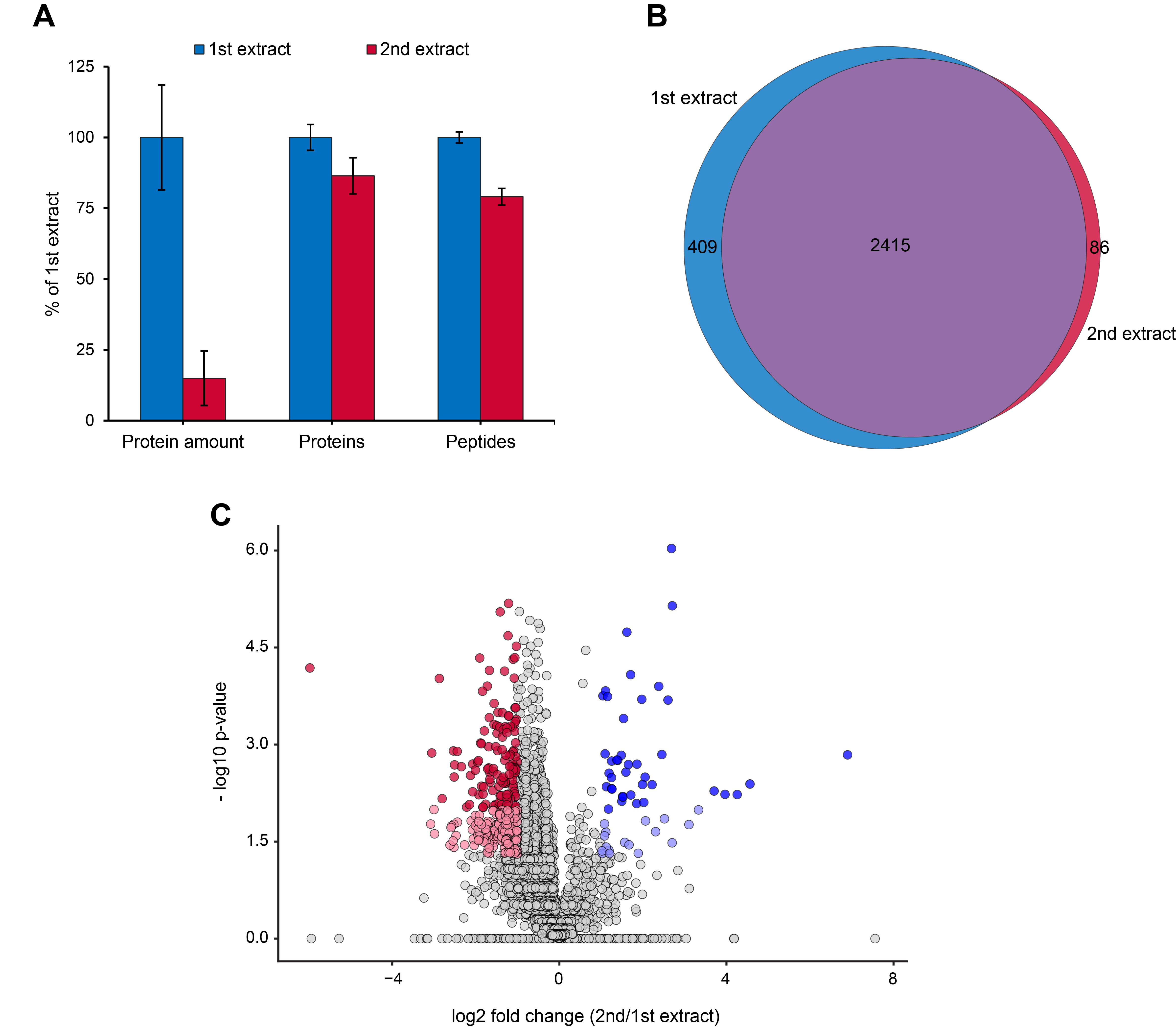
**

**Figure S2:** Re-extraction of residual FFPE tissue after first protein extraction with SDS buffer. (A) Extracted protein amount and identified protein groups (minimum 2 peptides per protein) and unique peptides obtained from two protein extractions from FFPE human substantia nigra (1 section, 4 µm) using SDS buffer. Data is shown as means ± SD (n=4) and as % of the 1st extract (100% = 45 µg protein, 2,419 protein groups, 19,158 peptides). (B) Venn diagram showing overlapping and unique numbers of identified protein groups in samples from each extraction. (C) Volcano Plot representing the log2 fold changes in protein intensities vs. the negative log10-transformed p-values (n=4; two-tailed, equal variance t-test) of the second extract vs. the first one. Proteins with fold changes ≥2 (blue) or ≤2 (red) and p-values ≤0.01 (dark) or ≤0.05 (light) are displayed in colour.


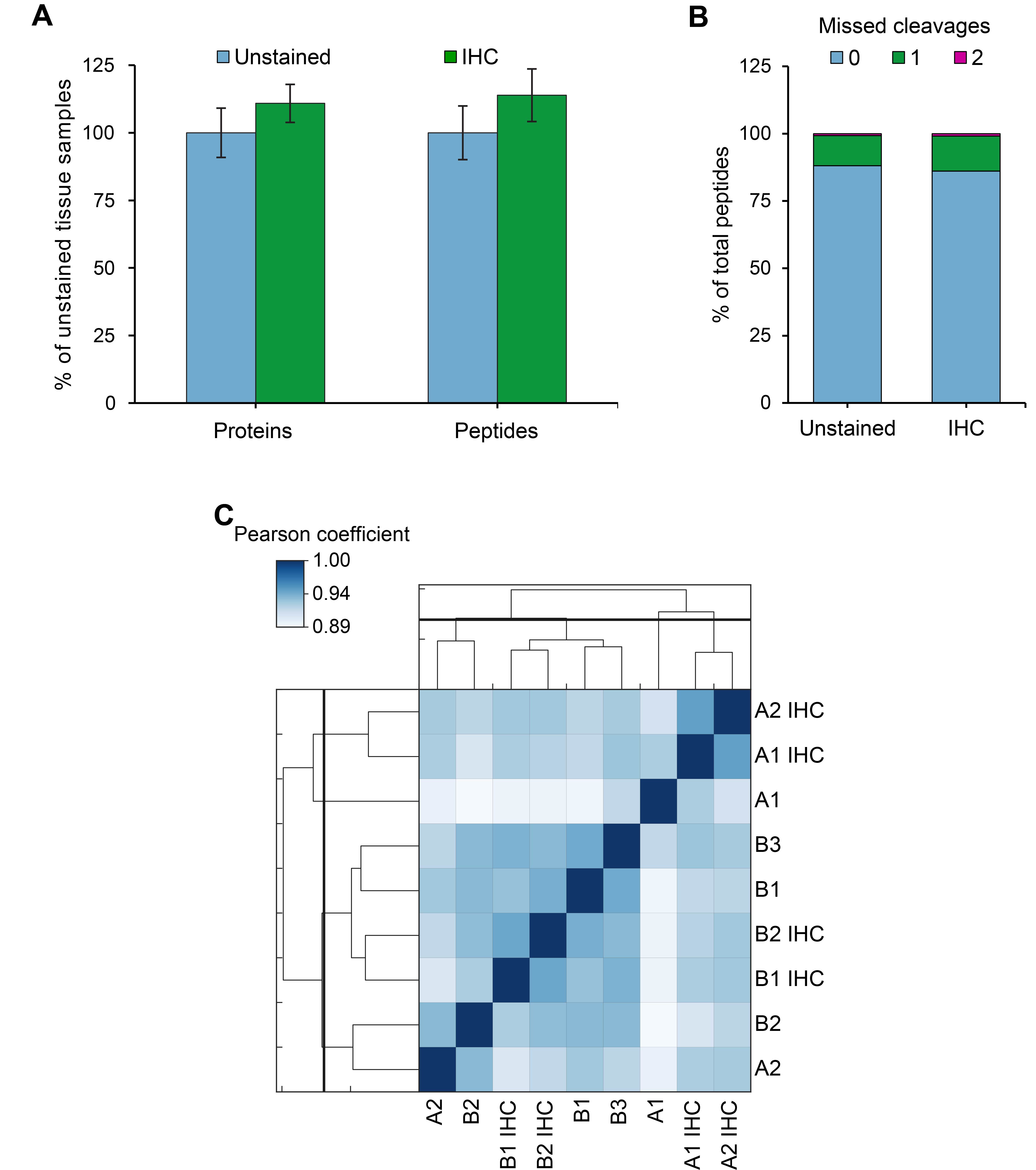


**Figure S3:** Influence of immuohistochemical (IHC) staining on proteins in FFPE tissue. (A) Number of protein groups (minimum 2 peptides per protein) and unique peptides identified from microdissected FFPE human substantia nigra (3,000 cells). Tissue sections were either unstained or stained with rabbit anti-tyrosine hydroxylase pAb (1:500) using diaminobenzidine. Data is shown as means ± SD (n=4 (IHC) and 5 (unstained)) and as % of unstained tissue (100% = 1,138 protein groups, 5,886 peptides). (B) Fraction of peptides with zero, one and two missed cleavages. (C) Matrix representing the Pearson correlation of median-normalized log2 protein intensities from unstained and immunostained tissue samples.

**
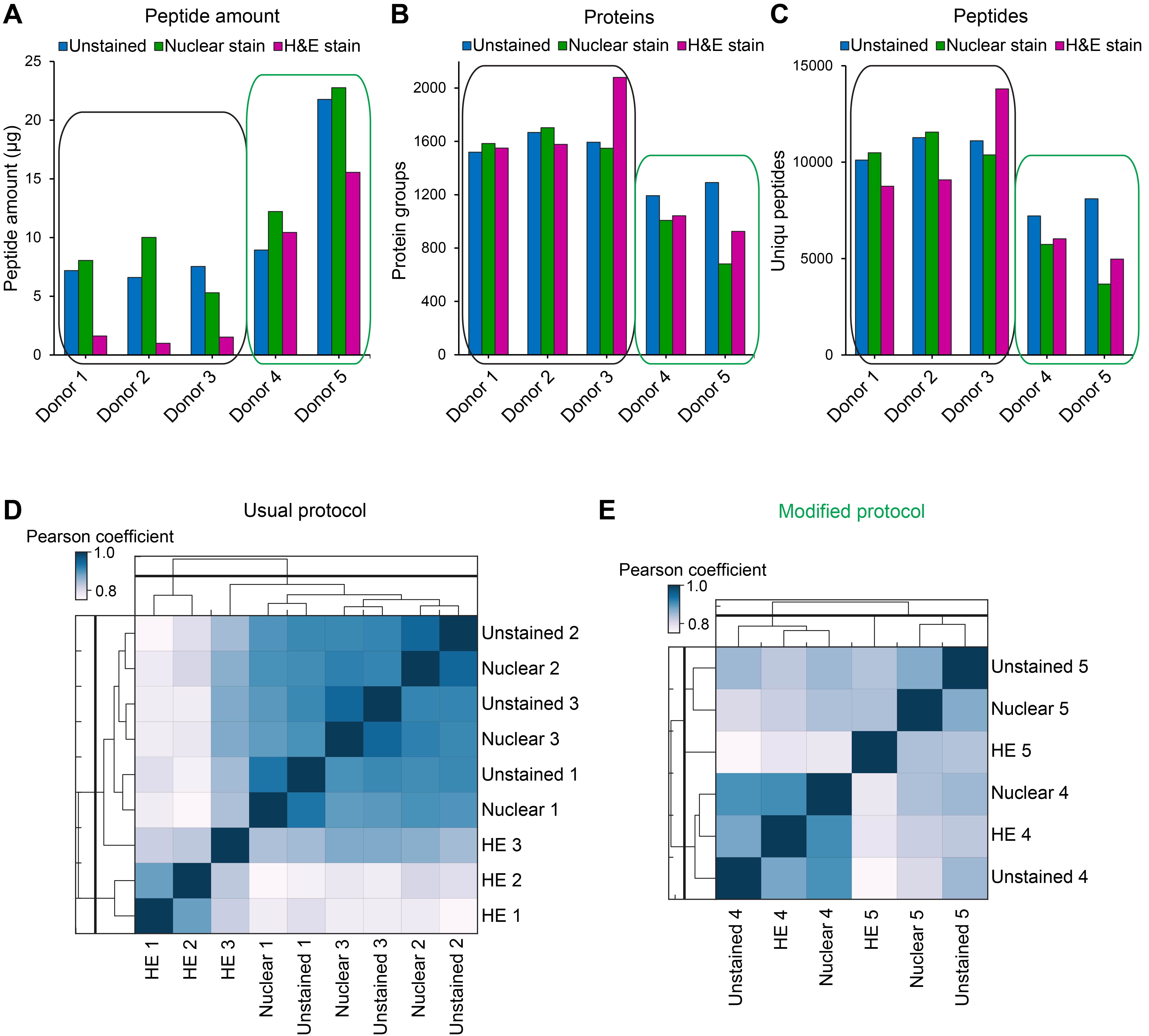
**

**Figure S4:** Influence of H&E staining on proteins in FFPE tissue. (A) Peptide amount obtained from unstained, nuclear (haematoxylin) or H&E stained FFPE human lung tissue (1 section, 10 µm). (B) Number of protein groups (minimum 2 peptides per protein) identified from unstained and stained FFPE tissue. (C) Unique peptide identifications from unstained and stained FFPE tissue samples. (D) Matrix showing the Pearson correlation of median-normalized log2 protein intensities from unstained and stained tissues prepared with the usual protocol (reduction/alkylation before SP3 protein clean up). (E) Matrix showing the Pearson correlation of median-normalized log2 protein intensities from unstained and stained tissues prepared with the modified protocol (reduction/alkylation after SP3 protein clean up).

**
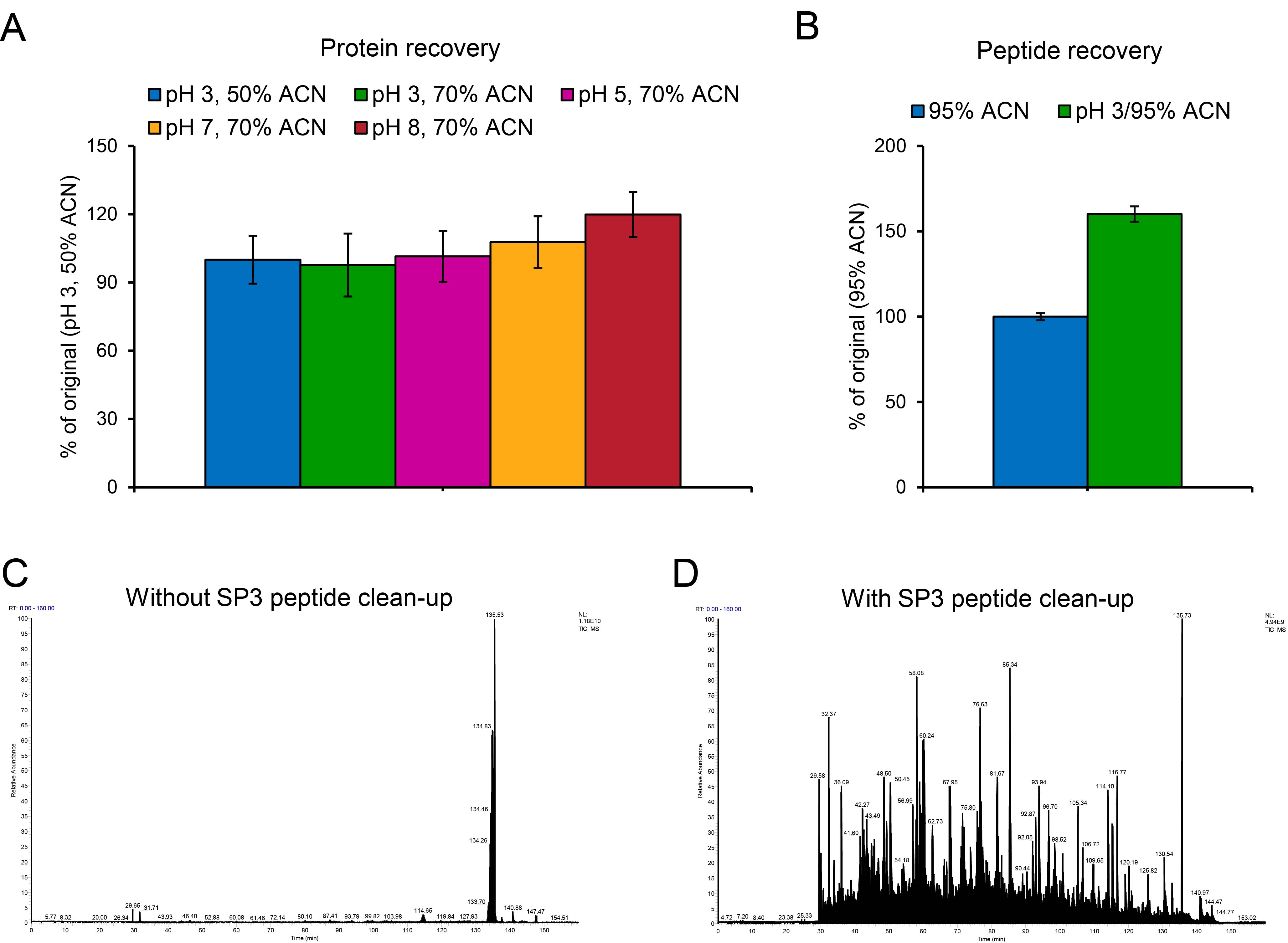
**

**Figure S5:** Optimization of the SP3 digest method. (A) Protein recovery after protein wash using different pH values and ACN concentrations of the washing solution. Data is shown as means ± SD (n=3) and as % of the original condition (pH 3/50% ACN). (B) Peptide recovery after peptide wash with and without acidification of the washing solution with formic acid. Data is shown as means ± SD (n=3) and as % of the original condition (95% ACN). (C) Chromatogram of sample prepared without peptide wash step after digestion. (D) Chromatogram of sample prepared with peptide wash step after digestion.

**
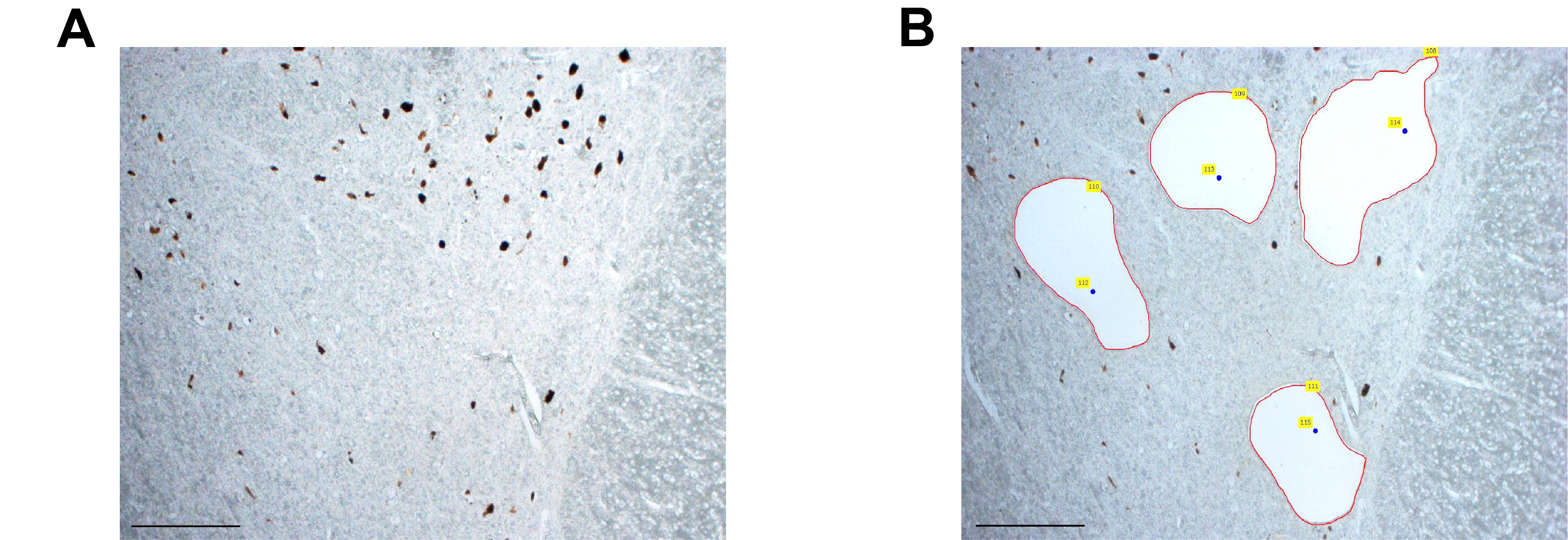
**

**Figure S6:** Microscopy images of FFPE human substantia nigra (10 µm thickness) (A) before and (B) after laser-capture microdissection. Scale bars 300 µm.

**
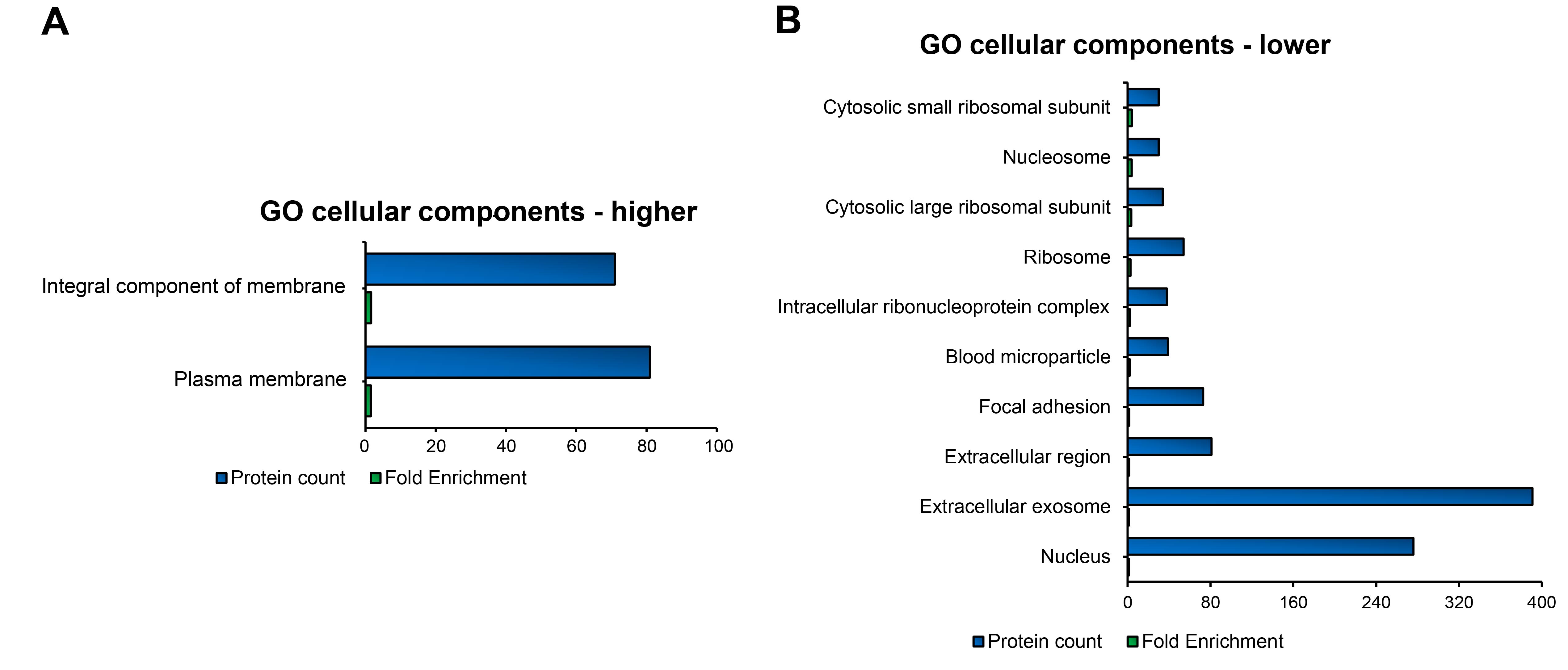
**

**Figure S7:** Comparison of microdissected tissue samples with intact sections. (A) GO cellular components enriched in microdissected substantia nigra (~3,000 cells) compared to intact sections (Benjamini-corrected p-value <0.01, protein count ≥10). Brain-specific proteome (proteins identified in both intact sections and microdissected substantia nigra) was used as background. (B) GO cellular components with lower intensity in microdissected substantia nigra compared to intact sections (Benjamini- corrected p-value <0.01, protein count ≥10). Brain-specific proteome was used as background.


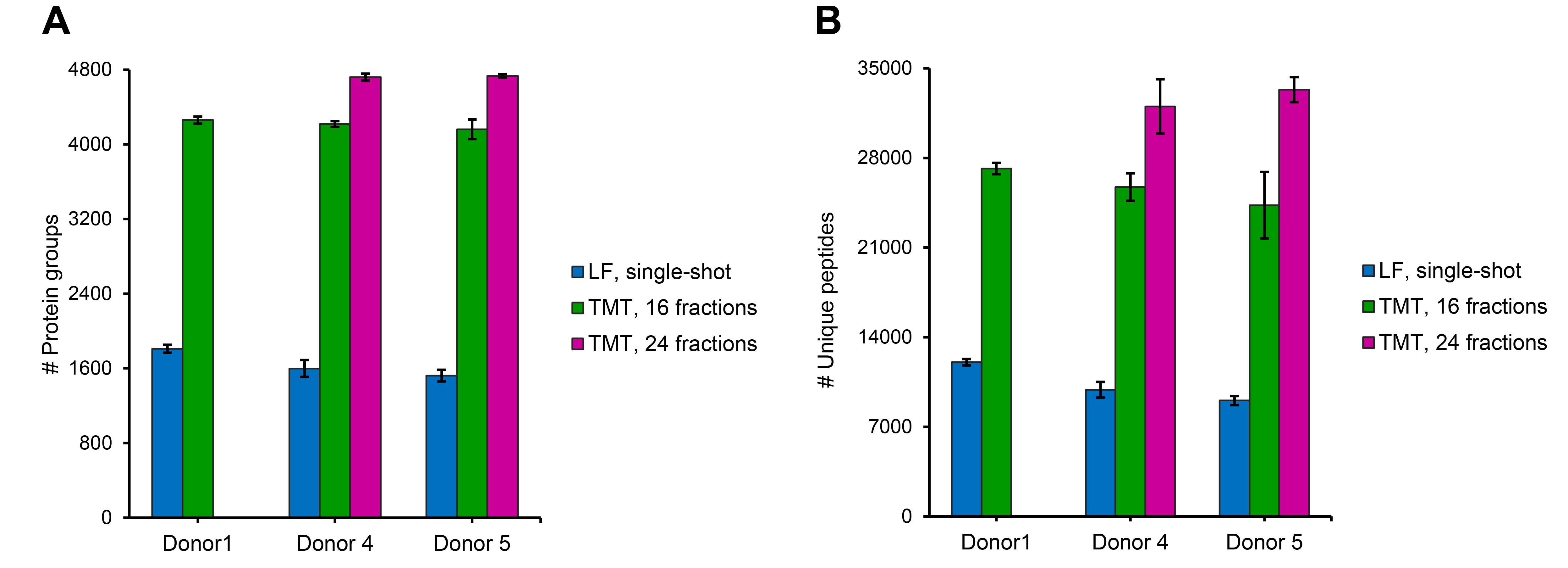


**Figure S8:** Comparison of quantified (A) protein groups (minimum 2 peptides per protein) and (B) unique peptides from label-free shotgun analysis and TMT-based analyses using fractions of microdissected FFPE human substantia nigra (3,000 cells). Data is shown as means ± SD (n=4).

**
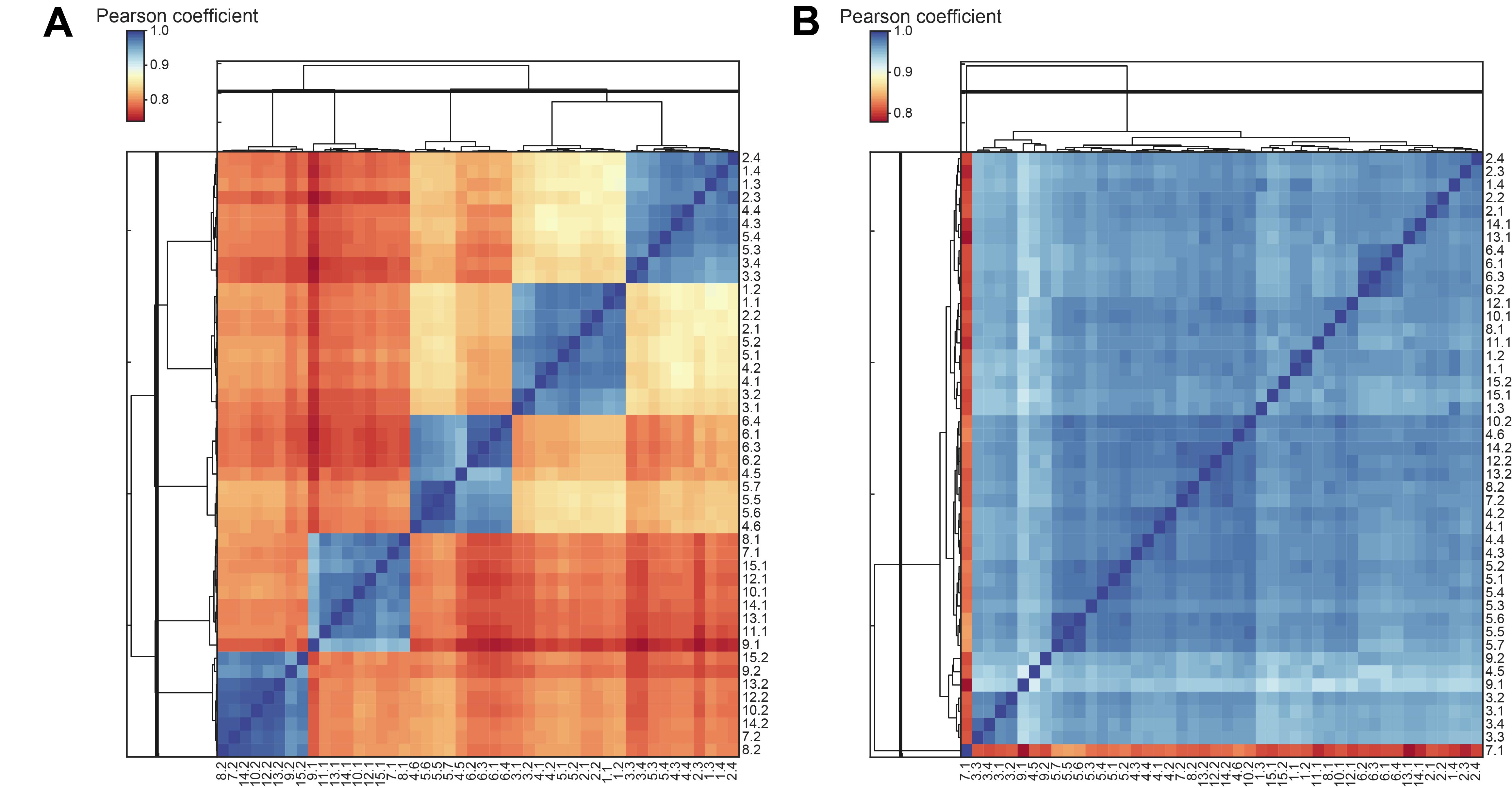
**

**Figure S9:** Normalization of protein intensities between TMT batches. (A) Matrix representing the Pearson correlation of log2 reporter intensities of all replicate samples analysed in five TMT batches. (B) Matrix representing the Pearson correlation of normalized log2 reporter intensities of all replicate samples analysed in five TMT batches.


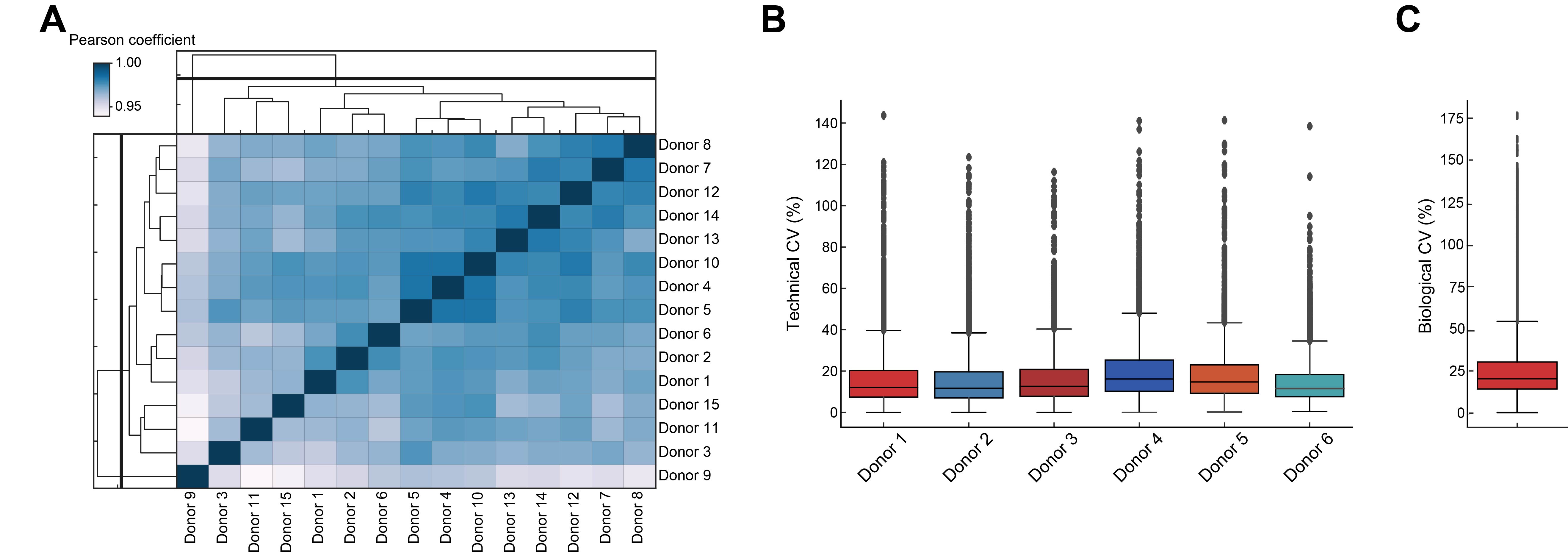


**Figure S10:** Variation of quantitative data. (A) Matrix representing the Pearson correlation between normalized log2 intensities (average of technical replicates) of different donors from human substantia nigra. (B) Box Plot showing the coefficient of variation (CV) of normalized protein intensities from technical replicates (n=4, 6 (donor 4) or 7 (donor 5)). (C) Box Plot showing the CV of normalized protein intensities from biological replicates (n=15).


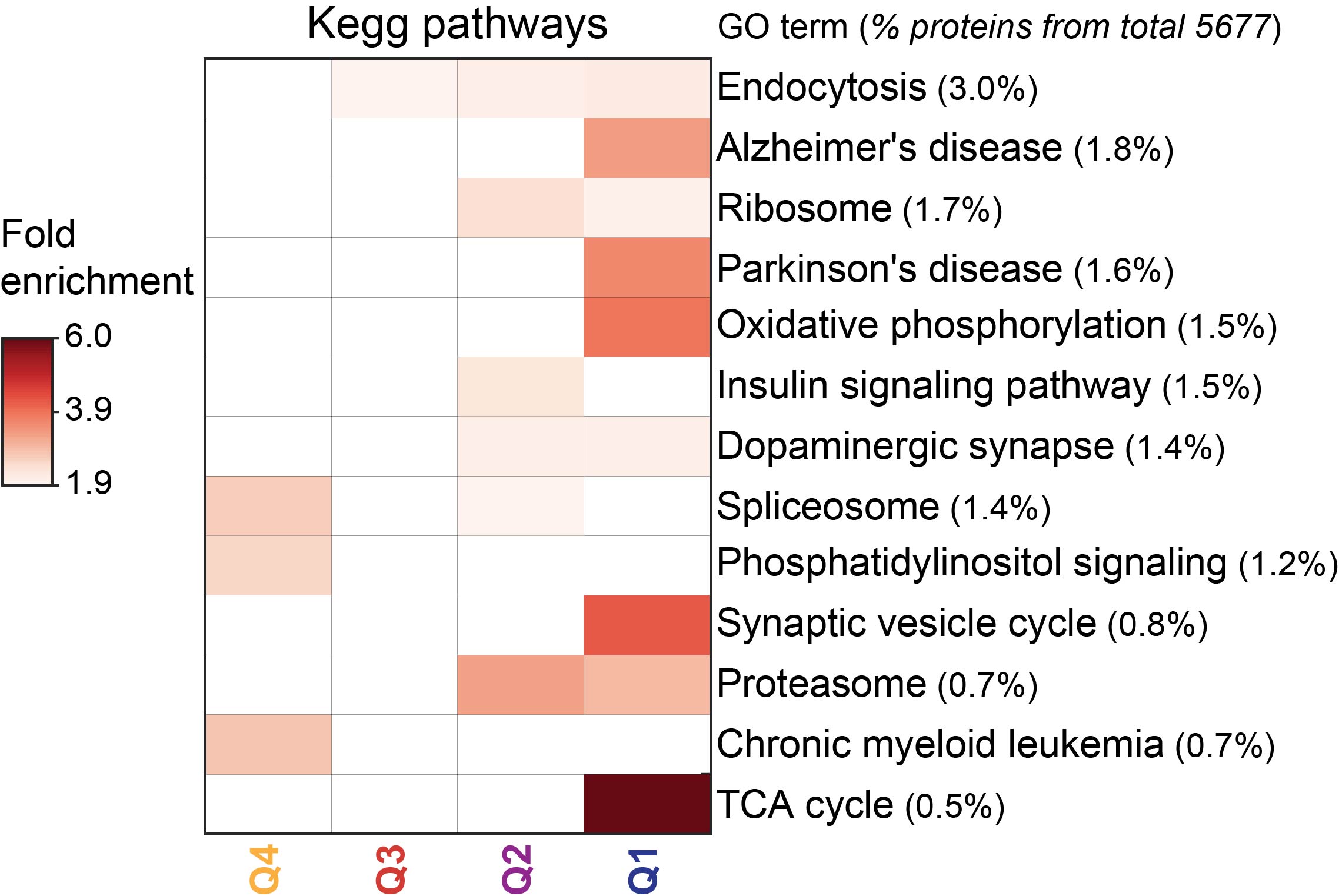


**Figure S11**: Functional enrichment of the nigral proteome. Heat map showing Kegg pathways enriched in each of the quartiles (Benjamini-corrected p-value <0.05, protein count ≥1%).
